# Developing a virtual reality and AI-based framework for advanced digital manufacturing and nearshoring opportunities in Mexico

**DOI:** 10.1038/s41598-024-61514-4

**Published:** 2024-05-16

**Authors:** Pedro Ponce, Brian Anthony, Russel Bradley, Javier Maldonado-Romo, Juana Isabel Méndez, Luis Montesinos, Arturo Molina

**Affiliations:** 1 Institute of Advanced Materials for Sustainable Manufacturing, Tecnologico de Monterrey, 64849 Monterrey, NL Mexico; 2 Department of Mechanical Engineering, Massachusetts Institute of Technology, Cambridge, MA 02139 USA

**Keywords:** Advanced manufacturing, Nearshoring, Training, Virtual reality, Artificial intelligence, Engineering, Information technology

## Abstract

The growing expansion of the manufacturing sector, particularly in Mexico, has revealed a spectrum of nearshoring opportunities yet is paralleled by a discernible void in educational tools for various stakeholders, such as engineers, students, and decision-makers. This paper introduces a state-of-the-art framework, incorporating virtual reality (VR) and artificial intelligence (AI) to metamorphose the pedagogy of advanced manufacturing systems. Through a case study focused on the design, production, and evaluation of a robotic platform, the framework endeavors to offer an exhaustive educational experience via an interactive VR environment, encapsulating (1) Robotic platform system design and modeling, enabling users to immerse themselves in the design and simulation of robotic platforms under varied conditions; (2) Virtual manufacturing company, presenting a detailed virtual manufacturing setup to enhance users’ comprehension of manufacturing processes and systems, and problem-solving in realistic settings; and (3) Product evaluation, wherein users employ VR to meticulously assess the robotic platform, ensuring optimal functionality and customer satisfaction. This innovative framework melds theoretical acumen with practical application in advanced manufacturing, preparing entities to navigate Mexico’s manufacturing sector’s vibrant and competitive nearshoring landscape. It creates an immersive environment for understanding modern manufacturing challenges, fostering Mexico’s manufacturing sector growth, and maximizing nearshoring opportunities for stakeholders.

## Introduction

Nearshoring involves a transnational organization establishing manufacturing, assembly, or sourcing processes in the country or continent where they intend to sell their products, reducing operational interruptions and ensuring the availability of supplies and efficient product delivery to the target market. Mexico has become an attractive option for companies looking to relocate their operations, particularly due to its proximity to the United States and the ease of exporting to this market under the United States–Mexico–Canada Agreement (USMCA). The Inter-American Development Bank (IADB) estimated that nearshoring could add $78 billion, $64 billion in exported goods^1^. Table 1 lists potential opportunities in nearshoring and shows the top 5 Latin American and Caribbean countries (LAC), in which Mexico is at the top position. *Quick wins* refers to the objective of securing 15% of the U.S. imports for the top 50 products that each LAC country was already exporting to the U.S. Within the context of *intra-LAC quick wins*, the goal is to attain a 15% market share of LAC imports for the top 50 products that each LAC country was exporting. In the case of *medium-term opportunities*, the aim is to achieve a 15% market share of U.S. imports for the top 50 products that LAC countries were exporting to Europe.

**Table 1:** Top five countries with potential opportunities of nearshoring for trade in goods (US$ millions)^1^.

Country	Quick wins - United States	Intra-LAC quick wins	Medium term opportunities	Total
Mexico	29,679.4	2628.2	2970.6	35,278.2
Brazil	4153	3144.3	546.8	7844.1
Argentina	890.7	1518	1497.8	3906.5
Colombia	1498.5	886.9	188.4	2573.8
Chile	665.8	516	641.1	1822.9

The IADB estimates that Mexico could generate up to $35.3 billion annually from nearshoring exports. Nevertheless, these imply several challenges, such as:

- Selecting the ideal business location (site selection), talent acquisition, and meeting trade agreement requirements.
- Reducing greenhouse gas emissions and creating a regulatory framework to facilitate digital commerce.
- Creating jobs, generating wealth, and offering hope for citizens seeking a better future.

Therefore, Mexico must develop a production ecosystem with a steady supply of raw materials, skilled labor, and improved logistics infrastructure and security^2^. Moreover, one of the sectors that most benefit from nearshoring is manufacturing. Thus, the future of manufacturing, especially within the context of Industry 4.0, needs a workforce proficient in a new set of skills, given the technological advancements and sustainability needs that are reshaping the sector globally. Several recent papers have embarked on the journey to identify and analyze these future skills, providing a roadmap for what will come in the manufacturing industry:

- Rodzalan et al. (2022) undertook a systematic literature review to explore the current and future skills that align with the demands of Industry 4.0, with a particular focus on Malaysia. This paper shows the impact of Industry 4.0 on the education and manufacturing industries, highlighting the imperative to bridge the gap between current and future skills of Industry 4.0^3^.
- Jurczuk and Florea (2022) identified existing gaps and future needs for digital design skills to support and comprehend the automation of business processes. A survey conducted in manufacturing companies across six European countries formulated a future-oriented digital design competence framework that addresses the requirements of process design and automation in the Factory of the Future^4^.
- Li (2022) emphasized that by 2025, 50% of all employees will need reskilling due to adopting new technology and that over two-thirds of skills considered necessary in today’s job requirements will change. The paper provides a blueprint as a reference for individuals to learn and acquire new skills and knowledge, suggesting that life-long learning should be integrated into an organization’s strategic goals^5^.
- Akyaziet al. (2022) developed an automated skill database for the manufacturing industry, particularly focusing on transversal occupations of this sector related to industrial symbiosis (IS) and energy efficiency (EE). The database incorporates each profile’s current and future skill needs, providing a valuable perspective on the future skills requirements resulting from industrial changes and sustainability needs^6^.

Incorporating artificial intelligence (AI) and virtual reality (VR) into this context, these technologies are becoming pivotal in enhancing skills and providing innovative solutions in the manufacturing sector. For instance, the *Umeed: VR Game Using NLP Models and Latent Semantic Analysis for Conversation Therapy for People with Speech Disorders* paper discusses using VR and NLP for training and skill enhancement through interactive, conversational scenarios, which can be extrapolated to manufacturing, where communication and interaction with systems and teams are crucial^7^. Moreover, the paper *Priority Experience Replay DQN for Training an Agent in Virtual Reality Game for Kids with Paraplegia* describes how an AI agent was created and then taught using the deep reinforcement learning deep Q-learning network (DQN) algorithm in a virtual reality game, providing feedback specifically suited to the individual’s skills and development. This concept of using AI to provide real-time, personalized feedback in a VR environment can be adapted to train manufacturing professionals in various tasks and operations, ensuring safety and precision^8^. Other type of approaches that involve VR focus in manufacturing, such as the *Automatic assembly simulation of product in a virtual environment based on interaction feature pair*, describes the automatic assembly simulation that is achieved from two main aspects: the interaction sequence provides the assembly order of the parts and the motion planning with a collision-free path for each part in a virtual environment^9^. *Testing the reliability of monocular obstacle detection methods in a simulated 3D factory environment* presents two proposed strategies for obstacle identification in an industrial context for driverless cars. The first method is based on convolutional neural networks (CNNs) for semantic segmentation, while the second method utilizes CNNs for depth estimation. Furthermore, a 3D virtual environment is developed in order to generate RGB pictures, ground truth segmentation, and depth images^10^. *A survey on HoloLens AR in support of human-centric intelligent manufacturing* offers a comprehensive review of the existing body of research on the application of HoloLens in industrial operations during the past several years. It aims to provide a detailed overview of the technical aspects of HoloLens-driven intelligent manufacturing^11^. Finally, *Towards user empowerment in product design: a mixed reality tool for interactive virtual prototyping* enables user participation throughout the first phases of the design process and facilitates the direct incorporation of their preferences, input, and specifications into the design models. The virtual environment and user interfaces provide the capacity for convenient customization to suit certain design environments^12^.

While the papers above provide a comprehensive overview of the skills and technological advancements in the global manufacturing sector, there is a noticeable gap in the literature regarding developing a virtual reality and AI-based framework for Advanced Manufacturing Education and Nearshoring Opportunities in Mexico. This could be an area ripe for exploration and research, considering the growing importance of nearshoring in developed countries and the potential of AI and VR in enhancing manufacturing education and operations. Developing a virtual reality (VR) and artificial intelligence (AI)-based framework for advanced manufacturing education and nearshoring opportunities, particularly in Mexico, can offer many advantages and significantly empower nearshoring endeavors.

Enhanced training and skill development can be achieved through VR, providing immersive training experiences and enabling workers to learn and practice skills in a safe, controlled virtual environment. At the same time, AI can personalize training programs, adapting them to individual learning curves and providing targeted feedback to enhance skill acquisition. AI algorithms can also improve operational efficiency by optimizing manufacturing processes, reducing waste, and enhancing productivity. VR is used for virtual prototyping and testing, reducing the need for physical models and enabling more rapid product development. Safety and risk management can also be enhanced with VR simulations that train workers to handle hazardous situations without real-world risk. AI predicts and prevents operational failures, enhancing workplace safety. Global collaboration can be facilitated through VR, enabling virtual collaboration and allowing experts worldwide to interact remotely with the manufacturing environment. In contrast, AI can facilitate communication and coordination among global teams, streamlining international collaboration.

**Figure 1:**
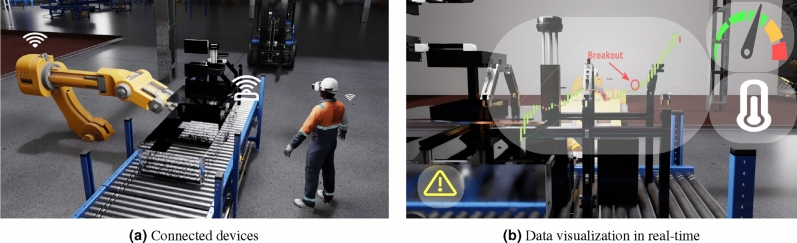
Industry 4.0: AI and VR.

Figure 1 illustrates an example of a virtual reality application in providing immersive training within a production line context (as shown in Fig. 1a). This visual representation showcases the capacity of virtual reality technology to create an environment where workers can undergo training in an immersive and controlled virtual setting. Moreover, it demonstrates how critical system information can be seamlessly integrated and presented within this immersive environment (Fig. 1b), thereby facilitating an effective learning experience for the workforce.

Additionally, a significant challenge is the maintenance of the line of production. Manufacturing systems are designed to be useful for years and experience progressive degradation, causing signs of oldness like noise, temperature changes, and pollution. Real-time sensing techniques and monitoring software enable continuous-time stochastic models for condition-based preventive maintenance. However, the side effects of degradation and the costs due to aging can lead to better-quality items with increased usage^13^. Likewise, existing maintenance based on time where maintenance scheduling literature is categorized into four main groups: ’Scheduling with a rate-modifying activity (RMA),’ ’Scheduling in a flexible window,’ ’Scheduling before reaching machine age,’ and ’Scheduling with a preventive maintenance policy.’ The first category deals with RMA, the second category involves advance interval scheduling, the third category requires scheduling before reaching a certain age, and the last category focuses on machine availability, considering two main objectives: (I) Maximizing machine availability, the goal of which is to maximize machine availability by determining the interval between preventive maintenance activities based on the reliability model of a system and the failure and repair data. (II) Maintaining a minimum reliability threshold assumes that the failure rate of a system tends to increase with time, making it susceptible to failures caused by age or wear^14^.

Likewise, Todescato et al.^15^ highlights the benefits of implementing reconfigurable and intelligent systems in production processes for sustainable manufacturing using digital technologies. These systems offer flexibility, rapid adaptation, customization capabilities, and integration of hardware and software flexibility. They also enhance sustainability by incorporating sustainability metrics into scheduling and decision-making processes. These systems can improve product quality and sustainability by integrating economic, environmental, and social dimensions, ensuring company growth. The holistic approach to flexibility and sustainability combines automation, information technologies, and artificial intelligence.

By strategically devising and adopting a virtual reality (VR) and artificial intelligence (AI)-infused framework for advancing manufacturing training, Mexico has the potential to substantially elevate its manufacturing capabilities. This concerted effort can position Mexico as an enticing nearshoring hub for international enterprises, particularly those originating from developed nations seeking a blend of skilled labor, technological prowess, and geographical proximity. The successful execution of such a strategy could potentially bolster the manufacturing sector within Mexico and foster a mutually beneficial rapport with its neighboring nations. This synergy, in turn, holds the promise of fostering economic expansion and technological progress across the region, filling a gap detected in the state of the art for nearshoring.

Likewise, including a case study enhances the practical relevance. The authors provide a concrete example of how the proposed framework can be implemented and its potential impact on assembling a virtual robot based on a physical representation in a virtual factory to estimate the production line and elements to produce, using a novel selection of elements to obtain the suitable final product to release that satisfy the needs on a specific environment.

## Proposed framework

Nearshoring entails outsourcing work to neighboring countries with lower wages to leverage geographic and temporal proximity, utilizing cultural, economic, and political linkages. Considering evaluation factors in distance, trade-offs, and supply costs is essential, integrating quantitative chain cost calculations and qualitative assessments^16^. Likewise, nearshoring is a business strategy involving outsourcing to neighboring countries and offers advantages over traditional offshoring. It emphasizes proximity, fostering better communication and cultural alignment. Cost savings are achieved without sacrificing quality, leading to higher customer satisfaction. Nearshore resources enhance project management, expertise, and risk management through local insights and explicit governance models^17^.

In response to the rapid expansion of the manufacturing sector, particularly in regions like Mexico, there is a pressing need to equip engineers, students, operators, and decision-makers with the knowledge and skills essential for success in this dynamic industry. To address these needs to face nearshoring, a comprehensive educational framework has been developed, which can be employed in areas such as immersive digital mockups for realistic design validation, realism at scale, VR multi-user conferencing, reduction of energy, large-scale world simulation, and virtual factories^18–20^. In this way, digital tools allow the development of a new way of interacting with manufacturing scenarios in an avant-garde way to improve processes, products, and services. In addition, it allows the incorporation of scaling considerations to expand since data are collected that provide information for decision-making in real-time.

**Figure 2:**
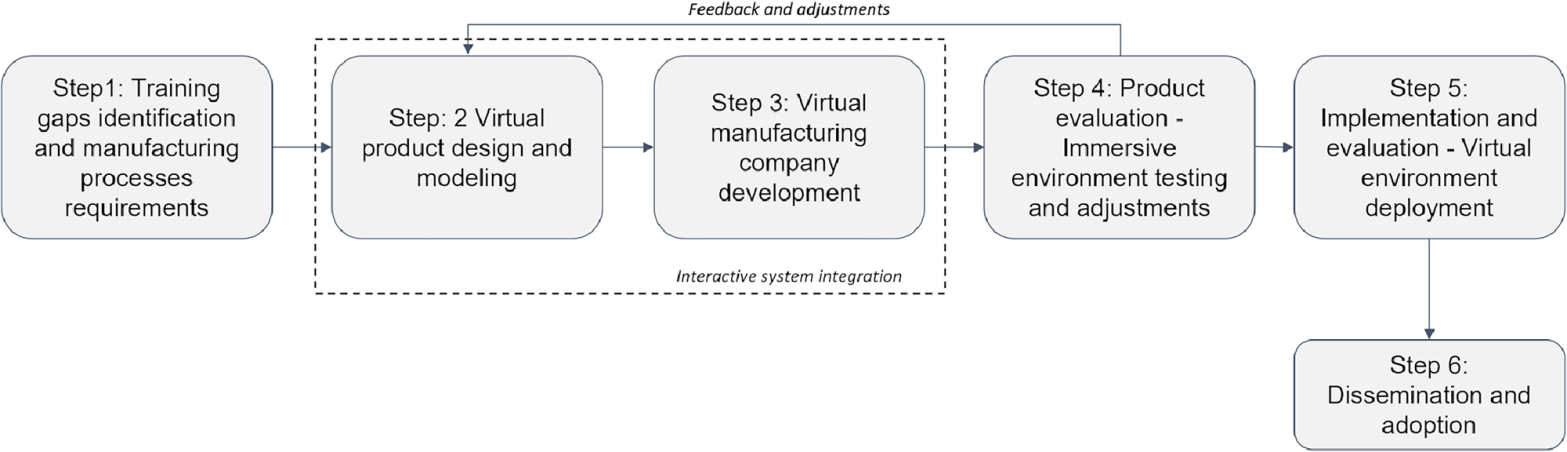
Proposed framework.

This framework leverages innovative technologies such as Virtual Reality (VR) and Artificial Intelligence (AI) to revolutionize the pedagogy of advanced manufacturing systems, offering an immersive and transformative learning experience. Figure 2 depicts the proposed framework that unfolds through a well-defined step-by-step process:

- Step 1: Training gaps identification and manufacturing processes requirements: This initial step involves conducting a thorough assessment of the target audience to identify specific educational gaps and requirements related to manufacturing processes. The insights gained from this assessment form the foundation for the framework’s development. Thus, the framework is introduced with a clear objective: to bridge training gaps and empower learners to excel in manufacturing processes. The integration of virtual reality (VR) and artificial intelligence (AI) is highlighted, as these technologies are at the framework’s core and play a pivotal role in the educational journey.
- Step 2: Virtual product design and modeling: This step empowers users with the skills and knowledge needed to comprehend, design, and simulate robotic platforms. The virtual design studio allows users to create, modify, and fine-tune robotic platform designs in an interactive VR environment. At the same time, the Simulation Lab provides insights into how different parameters and configurations impact performance and functionality.
- Step 3: Virtual manufacturing company development: This step provides a comprehensive understanding of manufacturing processes, equipment utilization, machinery maintenance, and inventory management. Users engage in hands-on activities within the virtual factory, interacting with production lines and applying problem-solving skills in realistic manufacturing settings.
- Step 4: Virtual product evaluation: Immersive environment testing and adjustment to evaluate the final product, a robotic platform, using VR technology. The users assess the robotic platform’s performance under different conditions and scenarios, and simulated customer interactions and feedback enable users to refine the product based on customer expectations. After this step, feedback and adjustments are performed based on the key takeaways and benefits. The framework’s role in preparing individuals and organizations for success in the manufacturing sector is emphasized. Continuous updates and enhancements ensure the framework remains agile and adaptable to evolving industry trends and technologies.
- Step 5: Implementation and evaluation: This step outlines how educational institutions or organizations can implement the framework, including technical requirements and instructor training. A plan for evaluating the framework’s effectiveness, including assessing user learning outcomes and collecting valuable feedback for further improvements, is also provided.
- Step 6: Dissemination and adoption: Strategies for disseminating the framework to the target audience, such as marketing campaigns and outreach efforts, are developed. Educational institutions and organizations are encouraged to adopt the framework, with its potential benefits in enhancing education in manufacturing processes emphasized.

### Step 1: Gaps identification and manufacturing processes requirements

In the case of interacting with multiple robots, the social behavior and preferences of end-users are collected. This feature enables them to review and modify any unacceptable actions or behavior. Moreover, robots communicate with other social products to better understand end-user behavior, which in turn helps improve robot-user engagement. It is pertinent to note that traditional robot design approaches dismiss the social aspects of human–robot interaction. Positive social aspects boost the robot’s performance when operators are involved. Sometimes, it is necessary to collect end-user data to understand robot control behavior.

Likewise, Méndez et al. (2019); Molina et al. (2021) proposed using the S4 framework to design functional prototypes and provide user-friendly sensing, smart, sustainable, and social features^21,22^. They defined each *S* as:  

- Sensing (S1): the ability of a system to recognize occurrences, gather information, and assess variations by utilizing sensors that enable the observation of physical or environmental circumstances.
- Smart (S2): denotes the integration of physical components, intelligent elements, and connectivity enhancements, enabling a product to possess intelligence and the ability to connect with other devices.
- Sustainable (S3): Considers the social, environmental, and economic factors to produce balanced and optimized performance. Here, the social aspect pertains to how a product positively impacts people’s quality of life.
- Social (S4): refers to the ability to independently observe, record, analyze, and modify consumer behavior, as well as adapt its online/offline features to enhance its performance or market acceptance.

### Step 2: Virtual product design and modeling–robotic platform system design and modeling

Several robotic platforms have been developed and installed in the industry. For instance, Kumar et al.^23^ presented a meticulous design of a skid-steering mobile platform coupled with a Cartesian serial manipulator to execute various agricultural tasks, as detailed in their work. Their exploration into conceptual and component design using Solidworks, simulation on undulating terrains with ADAMS software, and the analysis of control mechanisms to scrutinize errors and performance provides a comprehensive view into the multifaceted nature of robotic design in agriculture. In a distinct approach, Kharzhevskyi et al.^24^ focuse on the pivotal aspect of wheel design for robotic platforms, especially those intended for reconnaissance in challenging terrains. Their work underscores the imperative of calculating design strength, ensuring minimal deformation, and maintaining the highest safety factor for wheel shaft and L bracket thickness, safeguarding the robotic platform’s robustness and reliability in demanding conditions. Transitioning to the industrial manufacturing domain, Zhang et al.^25^ illuminate the significance of high-fidelity simulation environments in evaluating industrial automation, presenting a simulation platform that meticulously reconstructs the behavioral patterns of real systems by incorporating the impact of robot dynamics into the control logic of industrial tasks. Their work accentuates the necessity of achieving high simulation fidelity, particularly in scenarios involving cooperative robots and modular manufacturing devices, to ensure accurate and reliable outcomes in simulated industrial manufacturing scenarios.

Moreover, the integration of digital twins and web services in robotic deburring within intelligent manufacturing is explored by Stan et al.^26^. Their development of a digital twin for a robotic deburring work cell, alongside process planning and robot programming, not only enhances the operational efficiency of the work cell but also introduces a new web platform that enables remote monitoring, triggers alerts for unexpected events, and facilitates control to authorized personnel. Furthermore, their exploration into energy consumption strategies and the results of energy-efficient motion planning, alongside signal-based scheduling optimization of the robotic deburring cell, provide valuable insights into energy-aware robotic manufacturing. Expanding the scope with additional recent works, Molotla et al.^27^ introduce a configurable hybrid integral manufacturing platform that utilizes an industrial robot arm for both subtractive and additive manufacturing processes, demonstrating the feasibility of integrating various manufacturing tools controlled by a single system. Kim and Park^28^ explore the application of robotic platforms in the garment industry, focusing on the automatic alignment and placement of fabric patterns, although further details are not available. Park et al.^29^ present an autonomous robotic bin-picking platform that combines human demonstration with a collaborative robot and YOLOv5 neural network model for object localization without requiring prior CAD models or training datasets. Lastly, , Formoso et al.^30^ introduce the Mobile Meta-Material Interior Co-Integrator (MMIC-I), a robotic assembler designed for ease of assembly and a simple design with a low number of unique parts, providing a solution for in-space assembly of large-scale space structures.

The contributions above highlight the remarkable strides in robotic platform system design and modeling within various manufacturing contexts. However, the rapid evolution of manufacturing demands, technological advancements, and the increasing complexity of production processes underscore the need to continue developing and innovating robotic platforms. The future of manufacturing hinges on the ability to create robotic platforms that are adept at navigating the complexities and variances within manufacturing environments and capable of adapting to evolving technologies and methodologies. This necessitates continuous exploration and development in the field, where robotic platforms are designed with a foresight of future manufacturing challenges and technological shifts. Moreover, integrating emerging technologies such as artificial intelligence, machine learning, and the Internet of Things (IoT) into robotic platforms will enhance their capabilities, enabling them to operate with increased autonomy, precision, and efficiency. Furthermore, the development of robotic platforms should also consider aspects of sustainability, energy efficiency, and adaptability to various manufacturing scenarios, ensuring their applicability and efficacy in future manufacturing landscapes. The ongoing creation and enhancement of robotic platforms remains paramount to ensuring that the manufacturing sector can meet future demands, embrace technological advancements, and navigate the challenges of the ever-evolving industrial landscape. The collective efforts of researchers, engineers, and industry professionals will be crucial in steering the direction toward innovative, efficient, and sustainable robotic platform designs in manufacturing.

**Figure 3:**
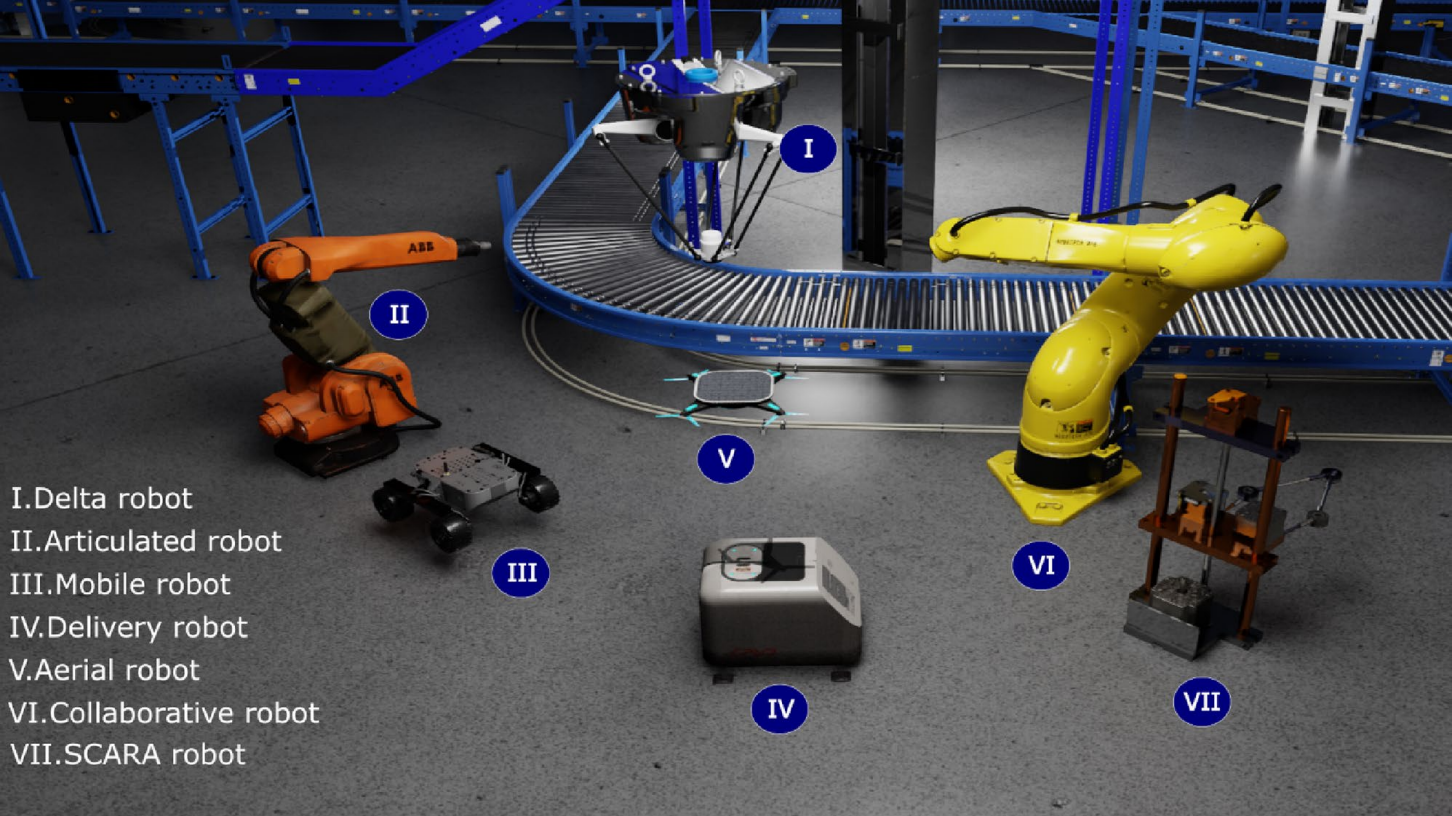
Robots in manufacturing in Industry 4.0.

In Industry 4.0, various types of robots have found applications in manufacturing processes. Each type of robot serves specific purposes and offers unique advantages in modern manufacturing. Figure 3 exemplifies seven different types of robots in manufacturing for Industry 4.0:

- (I) *Delta robots* These robots are widely used in pick-and-place operations, packaging, and high-speed assembly tasks. They are known for their exceptional speed and precision, making them suitable for tasks that require rapid and accurate movements, such as placing small components onto electronic circuit boards.
- (II) *Articulated robots* They have a wide range of applications in manufacturing, including welding, painting, material handling, and assembly. They are known for their flexibility and dexterity, with multiple joints and the ability to mimic human arm movements.
- (III) *Mobile robots* These are used for tasks that require autonomous navigation in dynamic environments. They are commonly employed in logistics, warehousing, and material transport within manufacturing facilities. They can also be utilized for inventory management and surveillance.
- (IV) *Delivery robots* Employed in the logistics and supply chain sectors. They are designed to transport goods within a facility, optimizing the movement of materials, components, and products. They can work autonomously or in collaboration with human workers.
- (V) *Aerial robots (Drones)* They have monitoring, inspection, and surveillance applications in manufacturing. They are used for assessing equipment, inspecting infrastructure, and capturing aerial imagery. Drones are particularly valuable in large-scale facilities or outdoor settings.
- (VI) *Collaborative robots (Cobot)* They are designed to work alongside human operators, fostering human–robot collaboration. They are used in tasks that require both automation and human oversight, such as precision assembly and quality control, and tasks that demand adaptability and fine motor skills.
- (VII) *SCARA robots* Selective Compliance Assembly Robot Arm (SCARA) robots are employed in assembly processes, material handling, and packaging applications. They are known for their speed, repeatability, and precision, making them suitable for tasks that demand high accuracy.

In Industry 4.0, these robots are often equipped with advanced sensors, vision systems, and connectivity features, allowing them to be part of the Internet of Things (IoT) ecosystem. They can communicate with other machinery and systems, enabling data-driven decision-making and enhanced automation. The choice of robot type depends on the specific manufacturing process, the need for precision, speed, flexibility, and the level of interaction with human workers. Manufacturers select the robot type that best suits their operational requirements.

### Step 3: Virtual manufacturing company development

**Figure 4:**
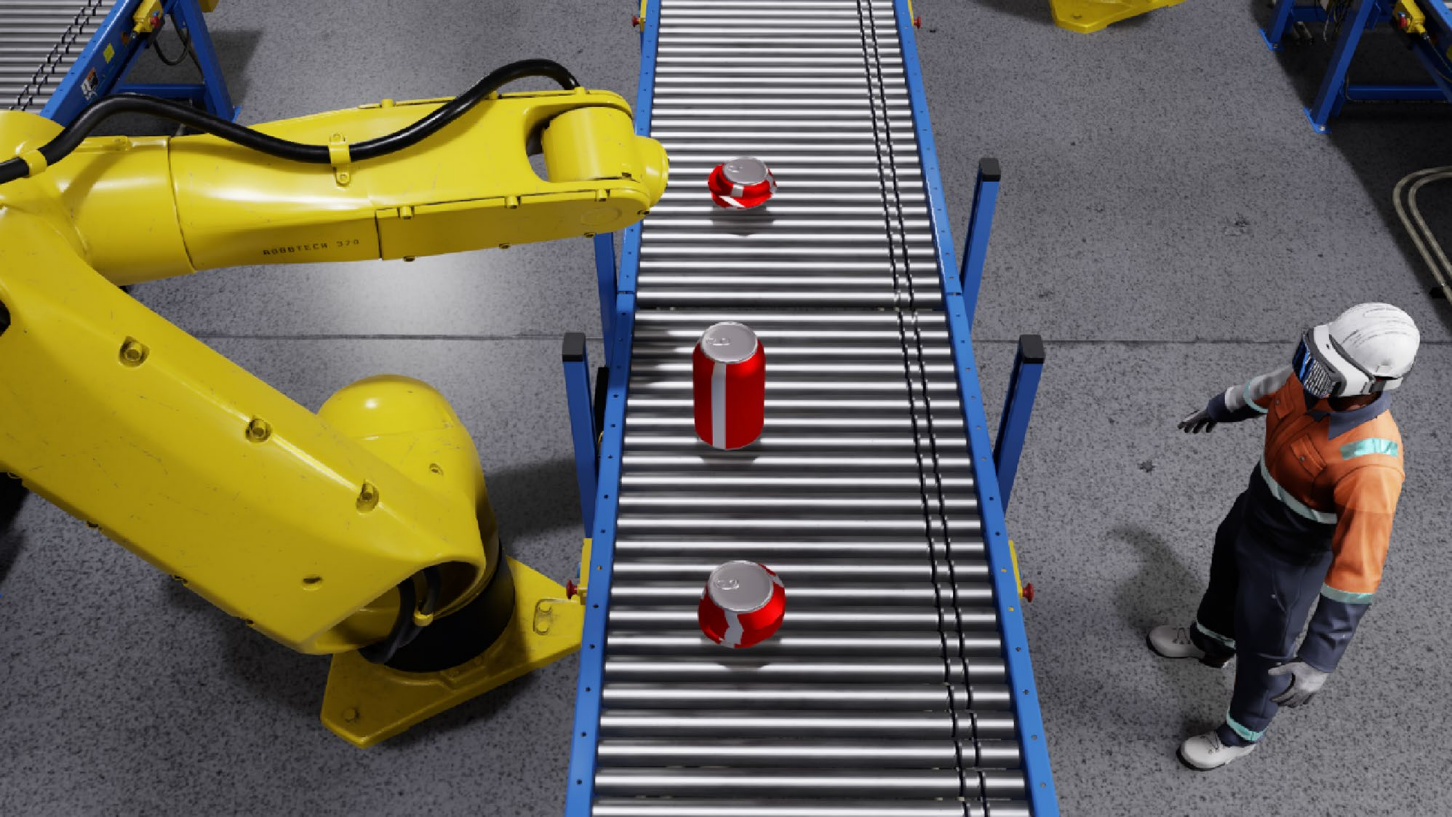
Virtual sampling for anomaly detection using computer vision.

In the study of virtual manufacturing companies, many innovative methodologies and technologies have emerged, each contributing substantively to the design and implementation of these digital entities. The integration of computational facilities and contemporary technologies, such as blockchain and machine learning, has been instrumental in amplifying product development and manufacturing capabilities. Vijayakumar^31^, in *On blockchain technology and machine learning algorithms in concurrent engineering*, shows the significance of integrating technologies like remote monitoring, artificial intelligence, machine learning, and blockchain in concurrent engineering applications, thus enhancing product development and manufacturing capabilities across various sectors. In a parallel, Serradilla et al.^32^ delineate a methodology that amalgamates data-driven techniques with domain knowledge in *Methodology for data-driven predictive maintenance models design, development and implementation on manufacturing guided by domain knowledge*. While sequential and ordered, this methodology encapsulates the essence of flexibility, ensuring that predictive maintenance (PdM) systems are meticulously tailored according to specific business and process characteristics, thereby intertwining with the concept of predictive maintenance in industrial settings. In a more specific instance of implementing virtual design in manufacturing, Starikov et al.^33^ introduce the concept of the Virtual Furniture Design Bureau (VFDB) in *Virtual Furniture Design Bureau: Distributed Design in Multi-Agent Environment Using Cloud Technologies*. The VFDB orchestrates a unified system environment for the distributed design of complex cabinet furniture products and manufacturing processes, employing object structural attribute models (OSAM) and a multi-agent approach, thereby facilitating distributed design and ensuring that the design process is managed and streamlined through a simulated multi-agent environment. Figure 4 shows an example of virtual sampling for anomaly detection using computer vision.

In a network-oriented perspective, Effendi and Efendi^34^ navigate through the design of a Virtual Local Area Network (VLAN) to manage broadcast and traffic on a computer network in *Design and implementation of virtual local area network at PT. eds manufacturing Indonesia*. This exploration into VLAN technology provides a lens through which the configuration of a virtualized computer network can be viewed, offering insights into managing network traffic and broadcasts in a virtual manufacturing setup. Marconi et al.^35^ propose an integrated shoe design and manufacturing approach in *A Digitally-enabled Integrated Approach to Design and Manufacture Shoe Lasts*. This approach, enabled by CAD/CAM technologies, integrates design and manufacturing phases and employs haptic technologies to interact with virtual models, simplifying subsequent planning and manufacturing operations. Zhou et al.^36^, in *Integration of Advanced Simulation and Visualization for Manufacturing Process Optimization*, likely delve into the integration of advanced simulation and visualization techniques to optimize manufacturing processes, providing a framework through which manufacturing processes can be visualized and optimized through advanced simulation techniques. In a parallel exploration of the Metaverse and virtual reality in manufacturing, Mourtzis^37^ in *The Metaverse in Industry 5.0: A Human-Centric Approach towards Personalized Value Creation*, delves into the alignment of the Metaverse concept with Industry 5.0, representing a digital ecosystem where collaboration fosters innovation and enhances productivity through the integration of technologies like AI, VR, and IoT. tegration of technologies like AI, VR, and IoT. Chu et al.^38^, in *An Exemplary Case of Industrial Metaverse: Engineering Product Demonstration Using Extended Reality Technologies*, present a framework that incrementally integrates virtual and real environments, developing VR, AR, and MR tools to support technical marketing, enabling real-time interaction for potential customers to evaluate industrial coolers without geographic and temporal restrictions.

Muthmainnah et al.^39^, in *Impact of Metaverse Technology on Student Engagement and Academic Performance: The Mediating Role of Learning Motivation*, explore the impact of Metaverse technology on student engagement and academic performance, revealing that student involvement can be positively impacted by the employment of Metaverse technology, which in turn improves academic performance. Wang et al.^40^, in *Nanomaterial-based flexible sensors for Metaverse and virtual reality applications*, delve into the advancements in nanomaterial-based flexible sensors (NMFSs), which can be tightly attached to the human skin or integrated with clothing to monitor human physiological information, provide medical data, or explore Metaverse spaces.

Finally, Li et al.^41^ mention that ensuring seamless integration and interaction between multiple platforms, devices, and data formats is crucial for achieving a ubiquitous semantic Metaverse with AI and VR technologies. This approach requires the development of intelligent algorithms that can allocate resources such as computational power and bandwidth in real-time based on user demand and distribution.

#### Maintenance

Genetic algorithms (GAs) are optimization algorithms based on natural evolution, transforming a population of individuals through mutation and crossover. They consist of a data structure, a fitness function, genetic operators, and a selection method for selecting more fit individuals for the next generation^42^.

Likewise, production scheduling and preventive maintenance (PM) planning are significant issues in the manufacturing industry. Equipment failures can disrupt schedules, while recommended intervals are often delayed to expedite production. Despite trade-offs, these activities are typically planned and executed independently^43^.

The model’s adaptability to various factory settings is a significant feature. It allows for the adjustment of parameters like the cost of preventive maintenance (CMP), the cost of failure (CF), equipment criticality (CE), and logistic costs (CL), along with the probability function. This flexibility makes the model applicable across different operational scenarios.

In a VR factory environment, this model provides a realistic simulation platform. It enables the analysis and visualization of different maintenance schedules and their implications, aiding in making informed decisions. The model identifies the most efficient maintenance strategy by balancing regular maintenance costs against equipment failure risks and potential expenses.

Comparing preventive and predictive maintenance mandates a comprehensive evaluation encompassing critical facets. A meticulous cost-benefit analysis is imperative to juxtapose implementation and operational costs against benefits such as downtime reduction, extended equipment life, and enhanced production efficiency. Despite upfront expenses, predictive maintenance offers a more favorable return on investment over time, driven by optimized resource utilization and reduced emergency repairs. Furthermore, its superior risk management capabilities bolster manufacturing stability^44,45^. Table 2 summarizes the information for each type of maintenance.

**Table 2:** Differences between preventive maintenance and predictive maintenance.

Characteristic	Preventive maintenance	Predictive maintenance
Scheduling	Scheduled at regular intervals	Based on real-time data and analytics
Costs	Predictable maintenance costs, prone to over-maintenance	Higher initial investments, but potential operational savings
Equipment Lifespan	It can prolong equipment lifespan	It can prolong equipment lifespan
Investments	Lower initial investments, but risk of over-maintenance	Higher initial investments in monitoring technologies and personnel training

Integrating this detailed cost function within the maintenance optimization model using a GA enhances the model’s precision and relevance in VR factory settings. It facilitates optimal maintenance planning, which is crucial for maintaining operational efficiency and reducing unnecessary expenses in various factory environments.

#### Sustainable and circular production

Although the design of the robotic system considers sustainability, it also highlights the consideration of sustainability in the production of the robotic system. Furthermore, It is essential to mention that the line of production must consider a circular economy approach to manage the waste generated by the process to create another type of value for the process or another type of process according to^46^, the waste is value for other business using strategies, such as durable design, maintenance, repair, re-use, re-manufacturing, refurbishing, and recycling^47^. In addition, the 9R framework promotes a circular economy approach by examining how products and materials can be used and reused at their highest value while minimizing waste and environmental impact^48^. Namely, the 9Rs are Rethink, Reduce, Reuse, Repair, Refurbish, Remanufacture, Repurpose, Recycle and Recover (from R1 to R9).

### Step 4: Product evaluation

Researchers have been exploring various methodologies and technologies to enhance the precision and efficiency of the evaluation process and products. A notable approach, as proposed by Bortolini et al.^49^. in their paper *A two-step methodology for product platform design and assessment in high-variety manufacturing*, involves a two-step methodology for product platform design and assessment in high-variety manufacturing, which utilizes a modified algorithm for solving the longest common subsequence (LCS) problem and k-medoids clustering. This methodology identifies the platform structure and assigns variants to the platforms, subsequently assessing them against various industrial and market metrics. Meanwhile, the emergence of virtual reality (VR) has opened new avenues in product evaluation, providing a unique, immersive environment where products can be evaluated in a detailed and interactive manner. Wang and Liu^50^ have delved into creating a virtual evaluation system specifically for product designing using VR. However, the specifics of their methodology are not detailed in the available information. Their paper is titled *A virtual evaluation system for product designing using virtual reality*.

On a similar note, a study by Palacios-Ibáñez et al.^51^ titled *Consumer Subjective Impressions in Virtual Reality Environments: The Role of the Visualization Technique in Product Evaluation* explored how different visualization techniques in VR environments can influence consumer subjective impressions during product evaluation. Their research, which involved evaluating different designs of a watering can in various VR settings, highlighted the potential perceptual differences that might arise when a product is assessed using different visualization techniques. Moreover, while evaluating a blood pressure monitor, Hinricher et al.^52^ investigated the effects of VR and the test environment on user experience, usability, and mental workload. Their findings, documented in *Effects of virtual reality and test environment on user experience, usability, and mental workload in the evaluation of a blood pressure monitor* , underscored that while VR evaluations should focus on objective criteria like user errors, subjective criteria such as user experience can be significantly influenced by VR. Another insightful research by Felip et al.^53^, titled *Touch Matters: The Impact of Physical Contact on Haptic Product Perception in Virtual Reality*, emphasized the impact of physical contact on haptic product perception in VR. Their work demonstrated that the means of allowing a product to be touched, which elicits a greater sense of presence, may positively impact evaluations of haptic features, especially for products with high haptic importance. In a different context, the VoicePrivacy 2020 Challenge, as documented by Tomashenko et al.^54^ in *The VoicePrivacy 2020 Challenge Evaluation Plan*, aimed to promote the development of privacy preservation tools for speech technology. Although not directly related to product evaluation, the methodologies and metrics developed during this challenge could offer insights into the development of evaluation methodologies in other domains.

### Step 5: Implementation and evaluation

Advanced data analytics and AI-driven feedback mechanisms are pivotal in this VR and AI-based framework. The system continuously collects and analyzes their interactions and performance data as users engage in the various modules. Artificial Intelligence algorithms, powered by machine learning, process this data to provide personalized feedback to each user. This feedback evaluates their progress and identifies areas where improvement is needed. For instance, AI algorithms can detect patterns in design choices or manufacturing process simulations and suggest alternative approaches based on best practices and historical data. This data-driven approach ensures learners receive tailored guidance, making their educational journey more efficient and effective. Furthermore, the system can offer insights into industry trends and emerging technologies by analyzing a vast dataset of manufacturing scenarios, equipping users with the latest knowledge to make informed nearshoring decisions. Data analytics and AI-driven feedback make this framework a dynamic and continuously evolving educational resource, aligning users with the ever-changing landscape of advanced manufacturing in Mexico and beyond.

### Step 6: Dissemination and adoption

Dissemination strategies are designed to spread the word about the framework, emphasizing its benefits and functionality. To achieve this, various marketing campaigns, both online and offline, will be employed. Additionally, workshops and seminars can be hosted, providing a hands-on experience to showcase the framework’s capabilities. Collaboration with educational institutions, industry partners, and government bodies will leverage resources and networks to enhance outreach. Online platforms and resources can be established, serving as hubs for information, discussions, and support. Furthermore, the framework’s flexibility, which allows customization to meet specific needs, will be emphasized, making it a more attractive choice for potential adopters. Success stories and case studies will be shared, demonstrating the positive impact of early adoption on education and manufacturing processes. Additionally, the commitment to continuous improvement will be reiterated, assuring adopters that the framework will be updated and aligned with evolving industry trends.

## Study case in design a virtual production based on a physical robot

This section describes the framework development applied in the virtual commissioning of a designed robot. The first stage consists of designing a virtual representation of a physical prototype, followed by a production line generated by the design features, and finally, the implementation stage in a particular environment. The COVID-19 pandemic prompted the development of new technology, including robots, to address public safety, clinical care, and work continuity. This robotic system is teleoperated through an intelligent glove, the robotic system is modular, this means that new attachments can be integrated according to the needs of each task to be solved. In addition, it has sensors to provide feedback to users to help them make decisions. Because it is a teleoperated system, the interface is a glove that indicates movements to interact with the environment in real time. The glove has a haptic feedback system to provide a more realistic experience of interacting with the environment, since in case of collisions, the user receives a response in vibration mode and also generates a stiffness to represent the forces of the environment on the robotic system. Furthermore, The S4 methodology, which has been used to create innovative products, is a low-cost robot^55^.

Four basic models, the function, the solution, the virtual, and the physical models, are determinant factors. Mechanical and software simulations can create a quick prototype. Bidirectional and visual communication are built into the robot to improve end-user-teleoperator engagement. Displayed eyes in robots foster a sense of social interaction, allowing people to interact with them rather than just seeing a robot. These features give the robot social qualities and responses to facilitate operator interaction. Robots can send messages to adjust driving circumstances; extra information appears if the operator drives the robot at high speed for a long time and the user adjusts the robot’s control.

### Step 1: Gaps identification and manufacturing processes requirements

S4 framework involves features that must be considered to develop sustainable products. Therefore, a virtual representation of the physical platform is developed to model the capabilities and describe the performance in time.

For each of the dimensions of the S4 framework, the following features are described: (1) Sensing, using a water sensor, proximity, infrared, camera, flow control, temperature, microphone, and RFID sensor. (2) Smart, including actuators such as ultraviolet light, BLCD wheels, embedded systems, and wireless connectivity. (3) Sustainable, low operating cost, low maintenance cost, intuitive interface, low energy consumption, safety, reduced environmental impact, and ergonomic assembly. (4) Social, the user acquires greater knowledge in their assigned tasks and the system is considered as a complement to the operator. These characteristics defined in the physical prototype have to be represented in the virtual model.

**Figure 5:**
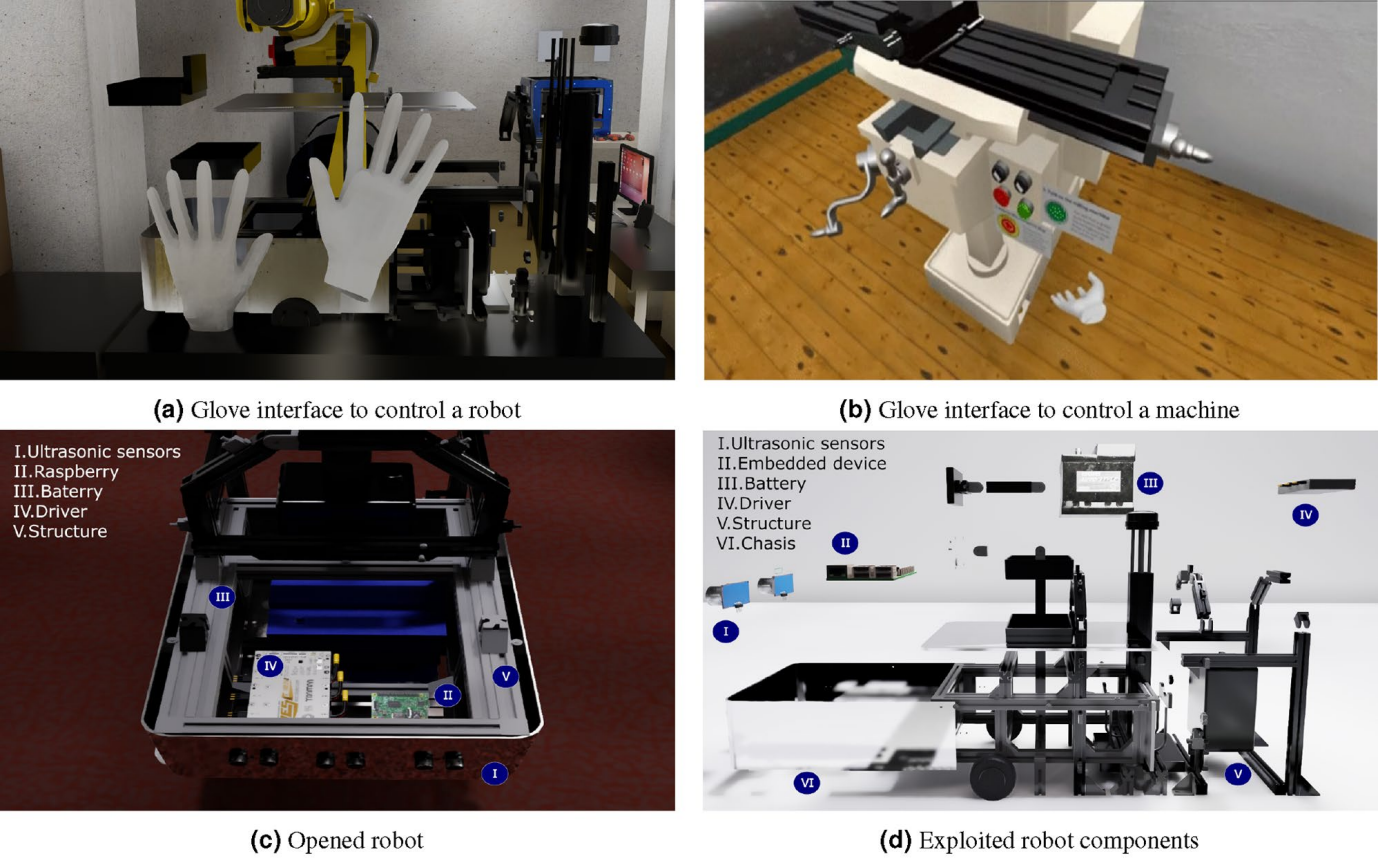
Virtual representation of the physical structure.

Figure 5 describes the modules based on the physical platform. For instance, Fig. 5a and b describe the interaction using a smart interface, such as a glove, which aims to control a robotic platform or a machine to transfer knowledge to training. Figure 5c shows a virtual representation of the physical robot shown the virtual components. Finally, Fig. 5d illustrates an exploited representation of each element.

### Step 2: Virtual product design and modeling

**Figure 6:**
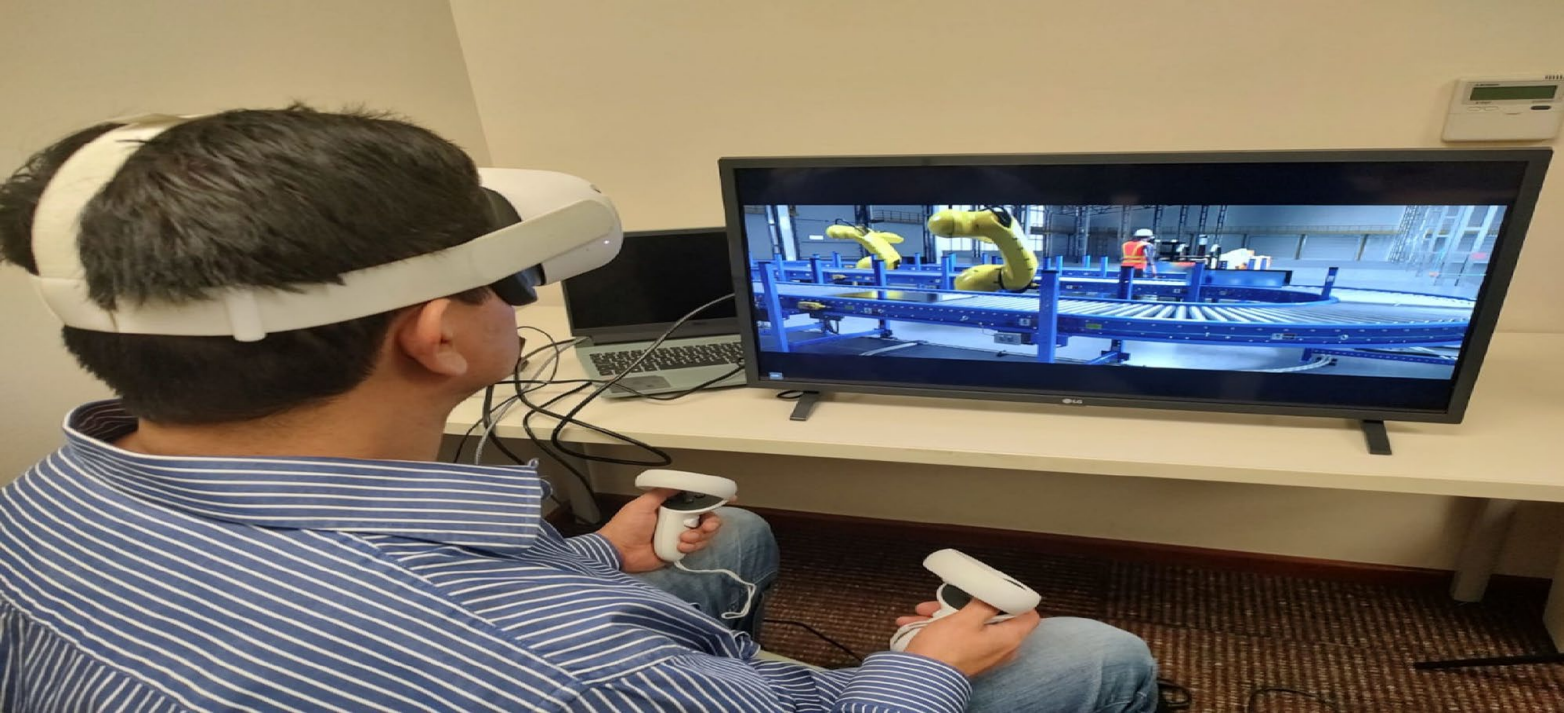
Virtual interaction between the user with the VR system.

The design of the robot is then scaled for production to meet customer demand. This consists of translating the design into production plans and assembly steps and then designing a production system capable of producing the robots at scale. The Bill of Materials and virtual representation of the physical design of the robot from the previous step are used to define the production and assembly steps. Figure 6 describes the interaction with the virtual system in real-time. Parts of the robot can be classified into commercial-off-the-shelf components, outsourced components, or manufactured parts. Each part’s manufacturing process is defined by considering the material, geometry, and mechanical properties. Machines are selected according to the specifications required to manufacture the parts.

**Figure 7:**
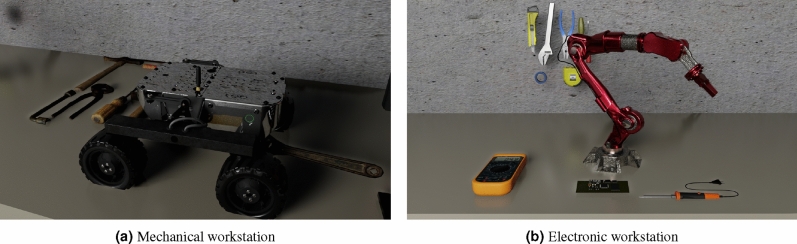
Virtual training workstations.

Since the design requires the virtual representation of the physical prototype, the workstations where workers develop the system modules are designed virtually, too. For instance, Figure 7a depicts the virtual representation of the structure and drive module of the physical prototype for the design and construction of virtual process improvements. Figure 7b illustrates the workstation for the electronics module. Each workstation has the tools and materials available in the physical world to perform similar tasks. The virtual representation workstations are required because the product design considers collaborative work where multiple specialists coexist in the design of the systems. Thus, a connection between the two environments must be maintained to reduce workflows. In addition, virtual workstations increase worker safety, improve the training of workers, reduce development time and cost, execute work tasks in parallel, and increase design quality.

### Step 3: Virtual manufacturing company development

Based on the virtualization of the production plan in the previous section, the production system can be described as a transfer line with the following stages: reception of raw materials and components, preparation of materials, assembly of the chassis and structure, installation of actuators and sensors, installation of the control system, partial tests, final assembly, and final tests.

A transfer line discrete event simulator can be run in the VR engine to simulate factory operations. The simulator takes in parameters from the production equipment manufacturers and the process plan developed in the previous stage. These include the mean time to fail (MTTF), mean time to repair (MTTR), cycle time distributions, and available buffer spaces between machines. The transfer line is modeled as a network of queues in the discrete event simulator. Through sampling cycle times on each station and failure and repair times from probability distributions, the simulation considers the variability of the processes. It can present production metrics such as overall equipment effectiveness, throughput rate, yield rate, lead time, etc. The simulation can be combined with additional data, such as demand forecasts and inventory policies, to increase the similarity to real-world production operations in a factory setting.

**Figure 8:**
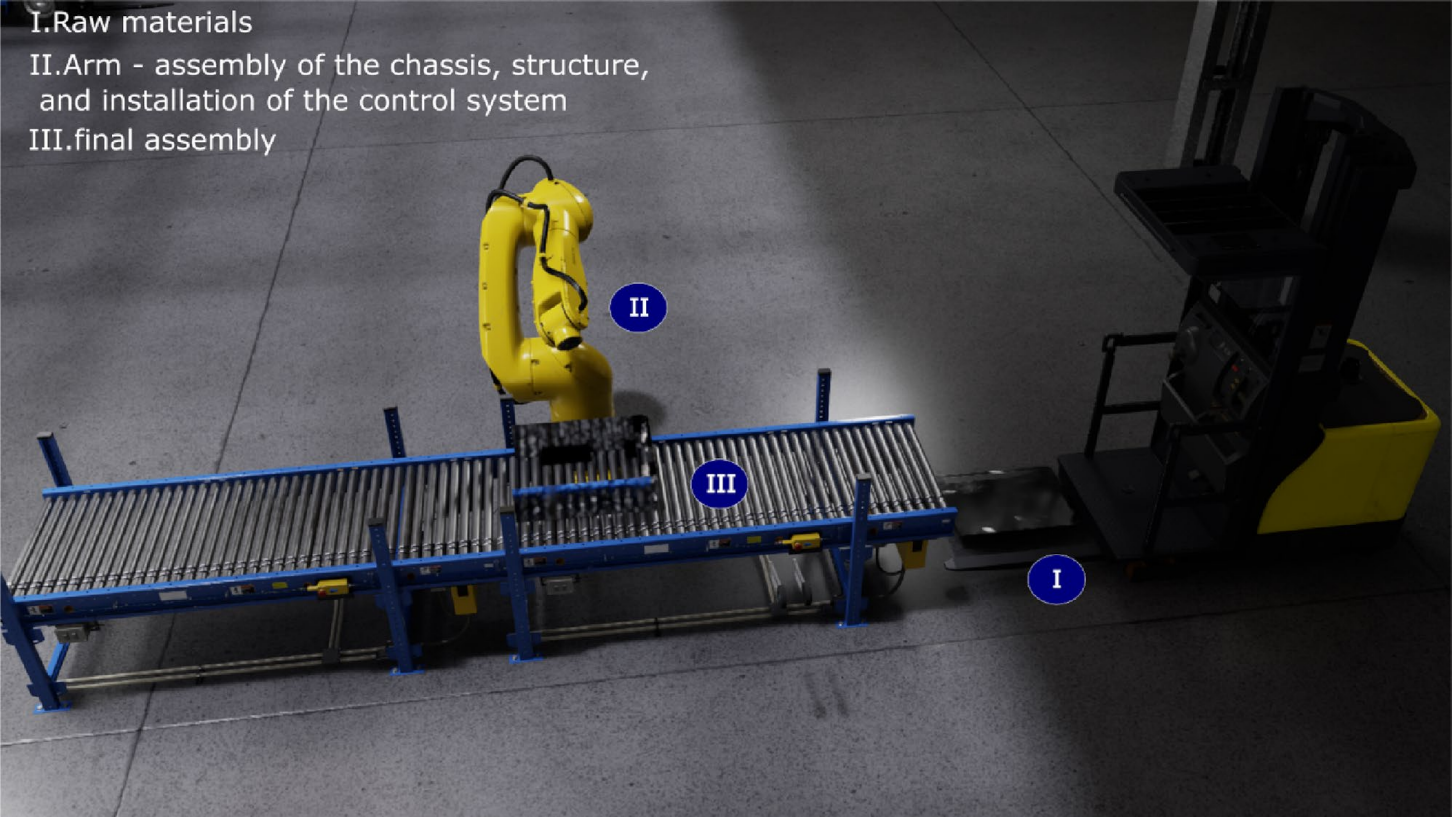
Partial line of production.

**Figure 9:**
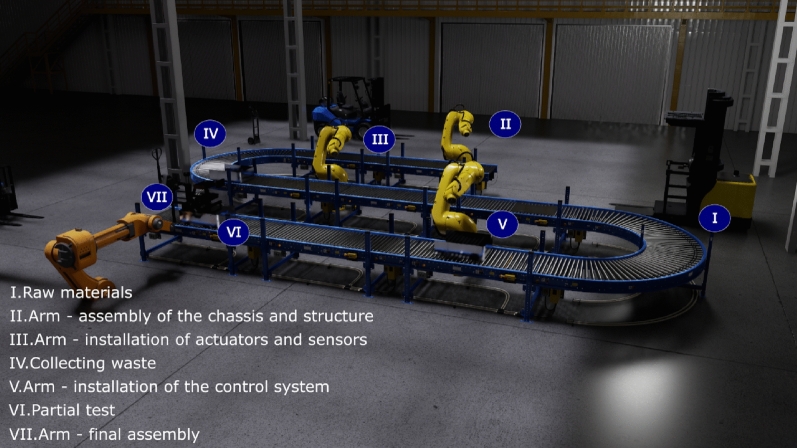
Final line of production.

**Figure 10:**
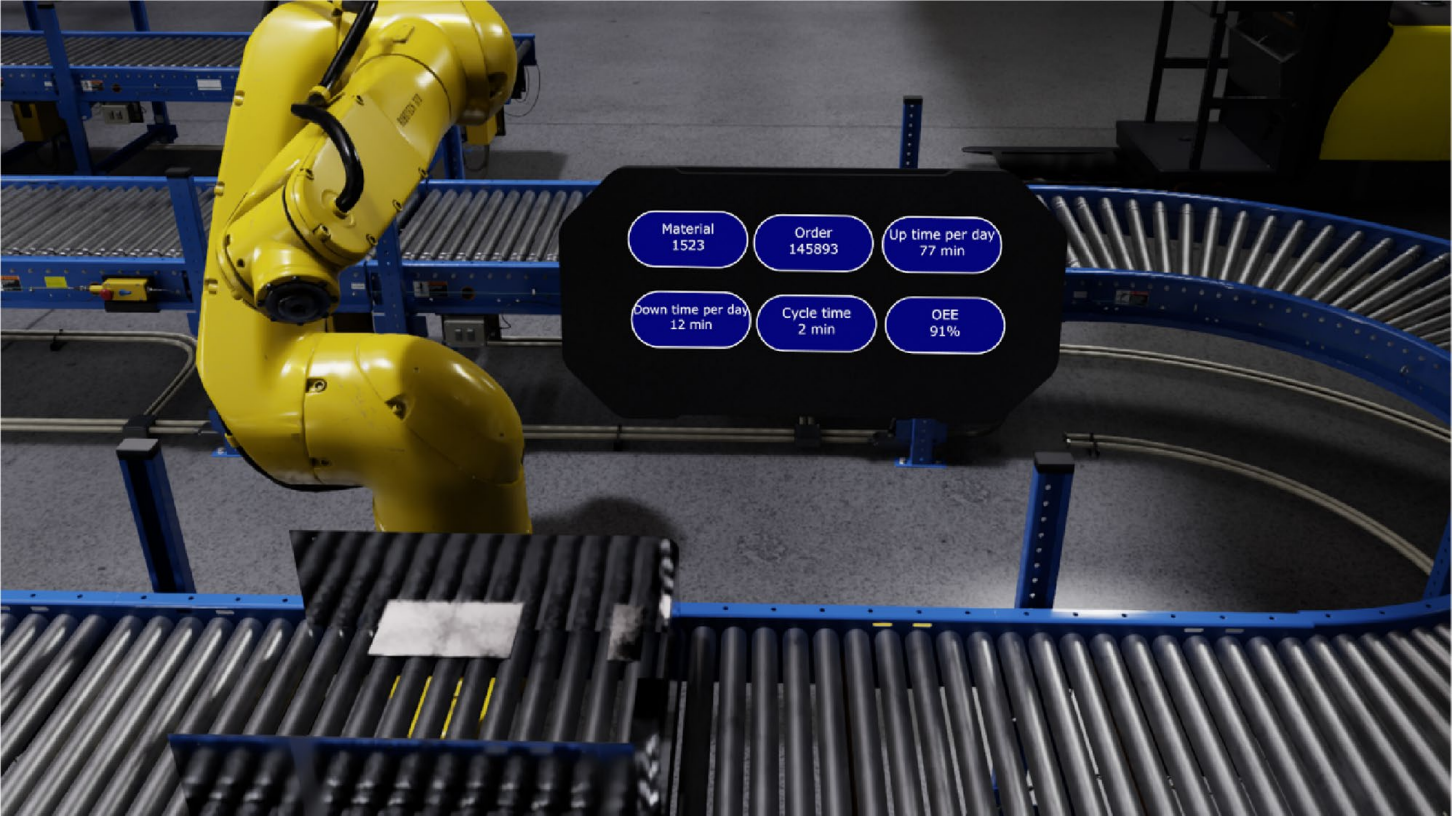
Dashboard to monitor the robotic platform.

One of the advantages of using virtual reality implementations is the distribution of the assembly stages of the prototype to simulate and analyze the efficiency of the stages. This proposal represents the stages above in a virtual environment designed to describe the production line. However, according to the assembly’s inventory, elements, and scalability, the line of production must adjust each time the product is designed. For instance, for a primary assembly, the elements involved in the line of production are limited due to the prototype’s elemental features, as shown in Fig. 8. Figure 9 depicts the cycle of all stages of product assembly production considering the scalability and each product module. The raw material location is transported by payload vehicles to the assembly line. There are four autonomous arms. The first one cuts the aluminum sheets. The second arm assembles the chassis and the structure. The third arm installs components, sensors, and system control installation. The last robotic arm evaluates partial tests by simulating the system integration. Figure 10 illustrates a dashboard that describes the features of the line of production to give essential information to the operator to decide in real-time.

**Figure 11:**
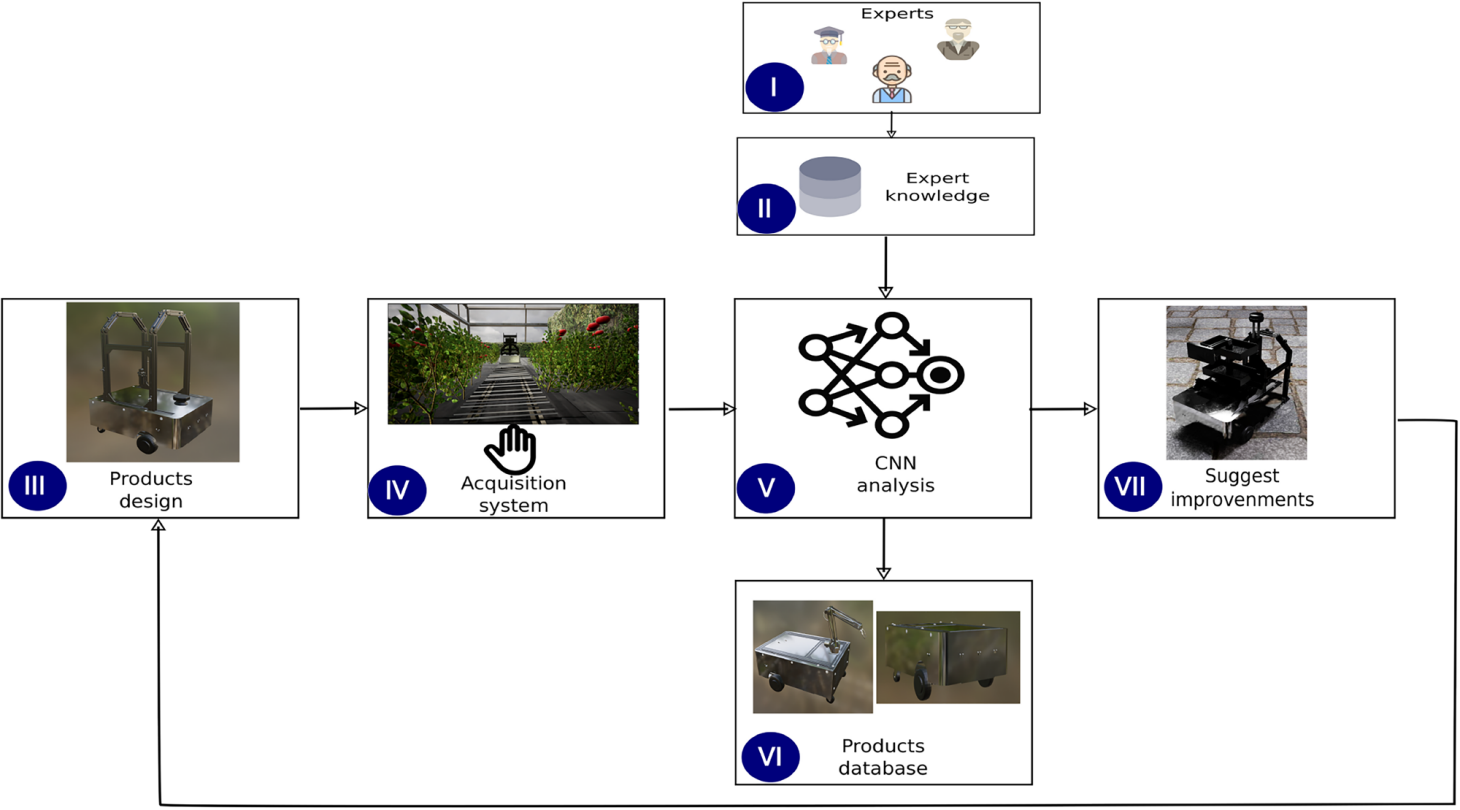
Approach for improving the product design process. Based on^56^.

To improve the performance of the robotic platform, beginning from a prototype, the features are adjusted according to the environment based on a knowledge database. Figure 11 describes the workflow to modify the features of the robotic platform. The first step is to collect data from experts (I). After that, a knowledge database is generated (II) to develop an initial prototype. Prototype (III) involves the main features according to the expert knowledge, and an environment is defined to evaluate the performance, collecting data generated by the robotic platform to make decisions (IV). A convolutional neuronal network (CNN) (V) estimates the performance and evaluates the generated value with the expected value. The criteria generated by CNN take the best features to adapt the current model to reduce the error in the evaluation stage (VI). The robotic platform’s features are modified for each iteration until a final design (VII). For this proposal, an agricultural environment is to evaluate the virtual system.

The procedural content generation (PCG) approach is employed to expand generative algorithms beyond constructive approaches used in the game industry to produce a limited range of content, emphasizing the integration of constraints into search-based PCG like a virtual prototype^57^. In this proposal, PCG operates based on four key constraints: (I) the weight of the material chassis, (II) the overall weight of the prototype, (III) torque in the attraction system, and (IV) the shape of assets to generate potential features of the virtual prototype.

Moreover, each constraint undergoes a normalization process and is subsequently multiplied by a constant determined through expert feedback. This systematic approach assigns weights to the most crucial features, aiming to derive the optimal combination during the search evaluation, prioritizing characteristics carrying the highest weighting.

Given the characteristics of a virtual prototype, a particular environment is created for proof-of-concept testing to evaluate the system. Each particular environment has specific features and conditions that are difficult to replicate in the real world for use in vision systems. Due to the requirements of the features of each sample, the virtual tools generate synthetic samples to train an object detection system to be implemented in the real world. However, they must have adequate detail to be detected in the physical world^58^. Synthetic samples are an alternative to increasing the diversity of samples in particular cases, such as in agriculture. The samples from the physical world of desired conditions, such as crop diseases, are complicated because they have to physically generate the crops with the conditions to collect them to obtain the samples.

**Figure 12:**
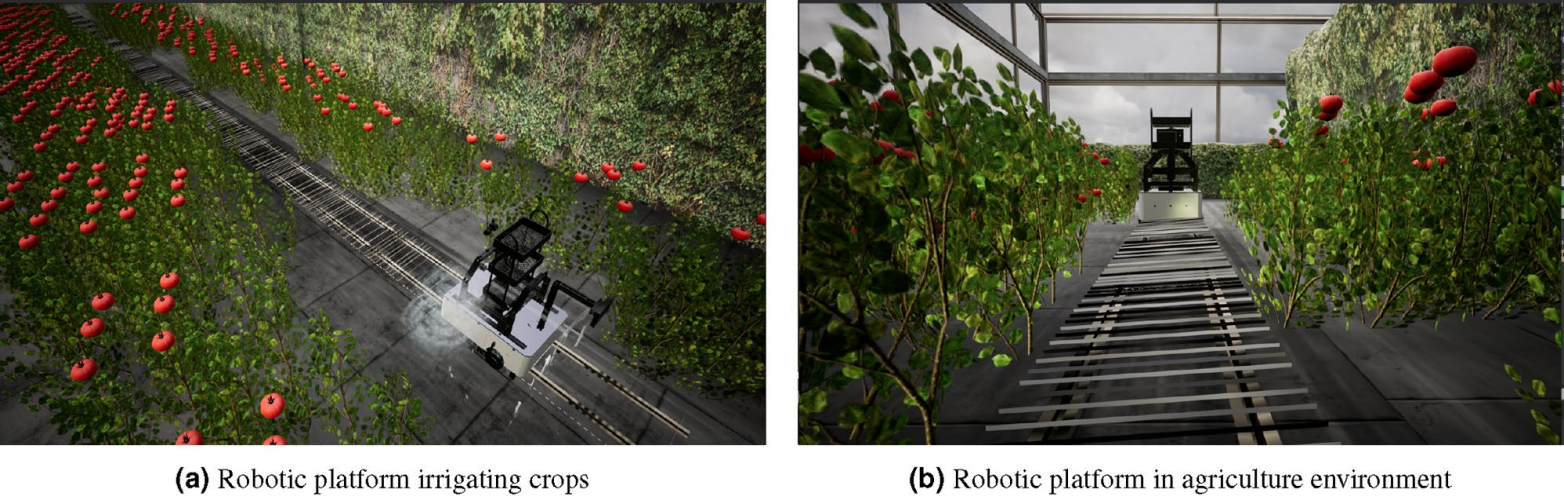
Robot platform in operation on virtual reality.

In this case, the test scenario is a greenhouse to detect anomalies in tomato crops in an environment that requires a rail to guide the robot’s path to move in a controlled scenario. The system has to move using a rail and detect anomalies in the crop to eliminate them and take tomatoes with better conditions. Then, an environment has been recreated that assimilates a tomato harvesting greenhouse with tomatoes with different characteristics to detect them in the system and remove them. In addition, there is a rail to help guide the system’s displacement. Since the robotic system is modular, the sprinkler is designed to irrigate water on its way in case it requires moisture in the soil. Figure 12 illustrates the robotic platform in the greenhouse environment.

#### Maintenance scenarios

Incorporating the detailed cost function into the Maintenance Optimization Model using a Genetic Algorithm in a VR factory setting provides a practical approach for scheduling maintenance. This model employs a genetic algorithm to determine the optimal timing for equipment maintenance by considering various influencing factors^59^. The key elements of this model, including the cost function, enable it to simulate factory conditions within a VR environment, enhancing decision-making capabilities.

The cost function (t, CMP, CF, CE, CL) is pivotal to this model. It defines the total cost of maintenance based on the probability of maintenance necessity, expressed through the function Equation 1. This probability function indicates that the likelihood of requiring maintenance increases over time, reflecting the equipment’s natural wear and tear. The total cost calculation combines preventive maintenance costs, represented by the costs associated with potential equipment failure. This formulation effectively balances the direct and indirect maintenance costs against the severity and probability of equipment failure expressed in Equation 2.

1 $$\begin{aligned} f(t)=1 - e^{-0.01 t} \end{aligned}$$

The cost function for maintenance represents the probability that maintenance is necessary or effective at time *t*. In a simplified model, this probability could increase with time, reflecting the increasing likelihood of equipment failure. However, this probability could be based on a factory model’s historical data, predictive analysis, and other operational factors.

- *CMP - cost of preventive maintenance* This is the direct cost of performing preventive maintenance. It would include labor, materials, tools, and any other expenses directly related to executing maintenance.
- *CL - logistic cost* This cost encompasses indirect expenses associated with performing maintenance, such as work planning and scheduling, spare parts inventory management, and any other logistic expenses.
- *CE - equipment criticality* This factor multiplies the cost of equipment failure based on the importance of the equipment. More critical equipment (i.e., those whose failure would have more severe consequences) would have a higher factor. This reflects the potential operational and financial impact of a failure.
- *CF - cost of failure* This is the cost of equipment failure if maintenance is not performed. It would include repairs, downtime, production loss, product quality impact, and other equipment failure costs.

2 $$\begin{aligned} Cost \ function(t) = f(t) * (CMP + CL) + (1 - f(f)) * CE * CF \end{aligned}$$

The total cost function aims to balance the cost of regular maintenance with the risk and cost of potential equipment failure. The optimization of this function would seek the point at which the combined cost of preventive maintenance and the risk-adjusted for the criticality of failure is minimized.

**Figure 13:**
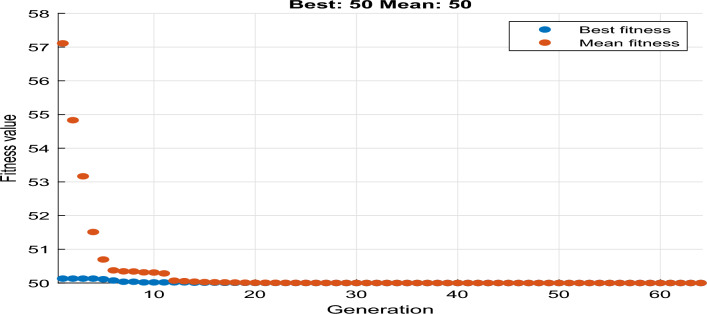
Performance for immediate maintenance.

**Figure 14:**
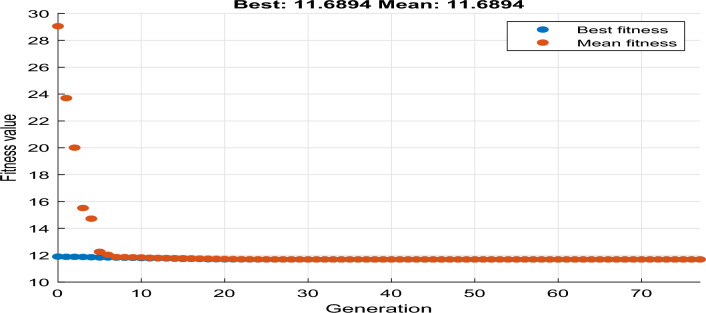
Performance for more than a year maintenance.

For instance, the cost function for the values with CMP =50, CF = 500, CE =0.1, and CL = 15 gives an optimized value of 50 and time of 0, which requires immediate maintenance, as shown in Fig. 13. On the other hand, CMP =5, CF = 50, CE =1.5, and CL = 5 give a minimized value of 11.68 and a time of 365 that requires maintenance for more than a year, as is shown in Fig. 14.

The maintenance model that has been suggested determines the characteristics of the assembly process based on a virtual model. It is important to note that the model operates in parallel to enable real-time behavior and is modular. This approach makes it easy to add new features or variables that need to be considered to provide a more realistic behavior.

#### Sustainable and circular production

**Figure 15:**
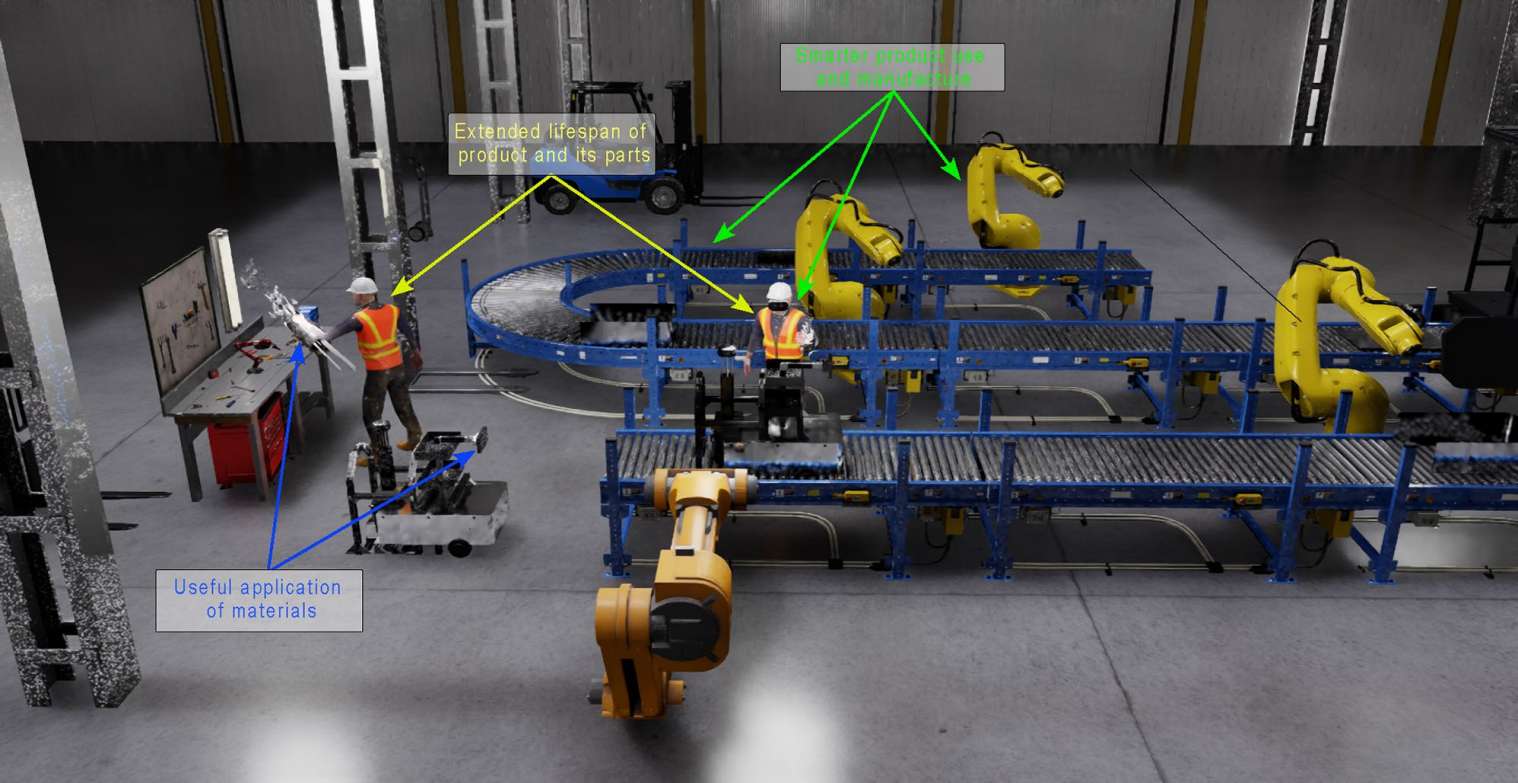
R9 framework groups in the virtual environment.

Circular production is characterized by various strategies outlined in the 9R framework^48^, each detailing actions to foster an environmentally friendly approach to virtual production line design. Morseletto et al.^60^ delineate three primary groups within this framework, each comprising specific actions to promote sustainability throughout the production process. The first group, “Smarter product use and manufacture,” encompasses strategies such as refuse, rethink, and reduce. These actions emphasize the importance of minimizing resource consumption and waste generation at the outset of the production process, thereby reducing the environmental impact of manufacturing activities. The second group, termed “Extended lifespan of product and its parts,” focuses on strategies to prolong the utility of products and their components. This group includes actions such as reuse, repair, refurbishment, remanufacture, and repurposing, which aim to extend the life cycle of products, minimize waste, and maximize resource efficiency through product reuse and maintenance. Finally, the third group, labeled “Useful application of materials,” centers on strategies related to the effective management of materials throughout their life cycle. This group encompasses recycling and recovery strategies, which aim to recover valuable materials from end-of-life products and manufacturing waste streams, closing the loop on material flows and reducing reliance on virgin resources. Manufacturers can significantly reduce their environmental footprint by employing these strategies in virtual production line design, promote resource efficiency, and contribute to the transition towards a more sustainable production paradigm. Figure 15 describes the distributions of each group of the sustainable strategies employed in the production line. Likewise, each group is described by each consideration below.

- Smarter product use and manufacture
  - In each main iteration, the production line is restructured to consider all the elements that allow the execution of the updated actions of the platform.
  - The number of assemblers is reduced, such as robotic arms, to which specific tasks were assigned to reduce time.
  - Take advantage of the infrastructure composed of robotic arms to execute actions in parallel.
  - Smart dashboards to assist the operator to improve the quality based in the collecting data sensors in real time using virtual environments.
- Extended lifespan of product and its parts
  - In each iteration, limited features are found, and the production line is adjusted to employ manual validation if required.
  - Use of the virtual environment to evaluate novel features based on the system simulation.
  - Use a virtual environment to modify and adjust the shape and distribution of the elements to assemble the product.
  - Employ previous designs to generate and describe improvements in terms of product performance.
- Useful application of materials
  - Identify points in the production line where raw material waste can be reintegrated or new value-added opportunities created. Use the waste material to create and adapt them to assets to expand the functionality of the robotic platform.
  - Adjust updated designs with previous considerations to include and improve the production line to reduce the impact on each iteration. For instance, use waste material to create tools like a material to cover the arms to secure the operations.

Moreover, the resources involved in the production line are identified. For instance, aluminum casings are treated as recyclable waste, enabling their integration as laminates. However, the focus is solely on reuse and waste reduction. An assessment of the energy expended in assembling the product must also be incorporated.

### Step 4: Product evaluation

As the robotic platform is teleoperated, it requires interaction with a user for its operation. Usability heuristics can be described as a product’s inherent attributes unaffected by extraneous variations in the input, such as (1) Visibility of system status, the method is trained using the best feedback that the product generates about what is going on. This feedback can be reached from the best products. (2) Match the system with the real world. The messages employ the user’s language (familiar words, phrases, and concepts) and follow fundamental conventions like symbols common to the product. Furthermore, (3) Helps users recognize, diagnose, and recover from errors; the method is trained for detecting error messages, which should be expressed in plain language (no codes), precisely indicate the problem, and constructively suggest a solution. Professionals comprehensively assess the usability criteria and corresponding descriptions within product design methodologies^56^. Therefore, heuristics-based product improvement features are enhanced through an approach based on deep learning to improve the product design process. This approach is composed of the opinions of several experts to create a knowledge base. Subsequently, it is evaluated through a deep convolutional neuronal network to determine the features stored in a product database. After selecting the product characteristics, improvements are proposed, and necessary iterations are carried out in a closed loop until the appropriate product characteristics are achieved.

The evaluation consists of describing the vehicle’s behavior in the test environment. The main objective of the evaluation is the interaction with the end user. For this reason, during the design of the robot, three iterations are carried out to suggest essential changes for the improvement of the platform. In each iteration, seven items are measured: user adaptability, maneuverability, position control, security, smoothness, path length, and short distance, which describe the performance of each user. Therefore, these proposed items describe particular tests to generate evidence to manipulate the virtual agent based on elemental movement. For each iteration, 30 persons used the system.

**Figure 16:**
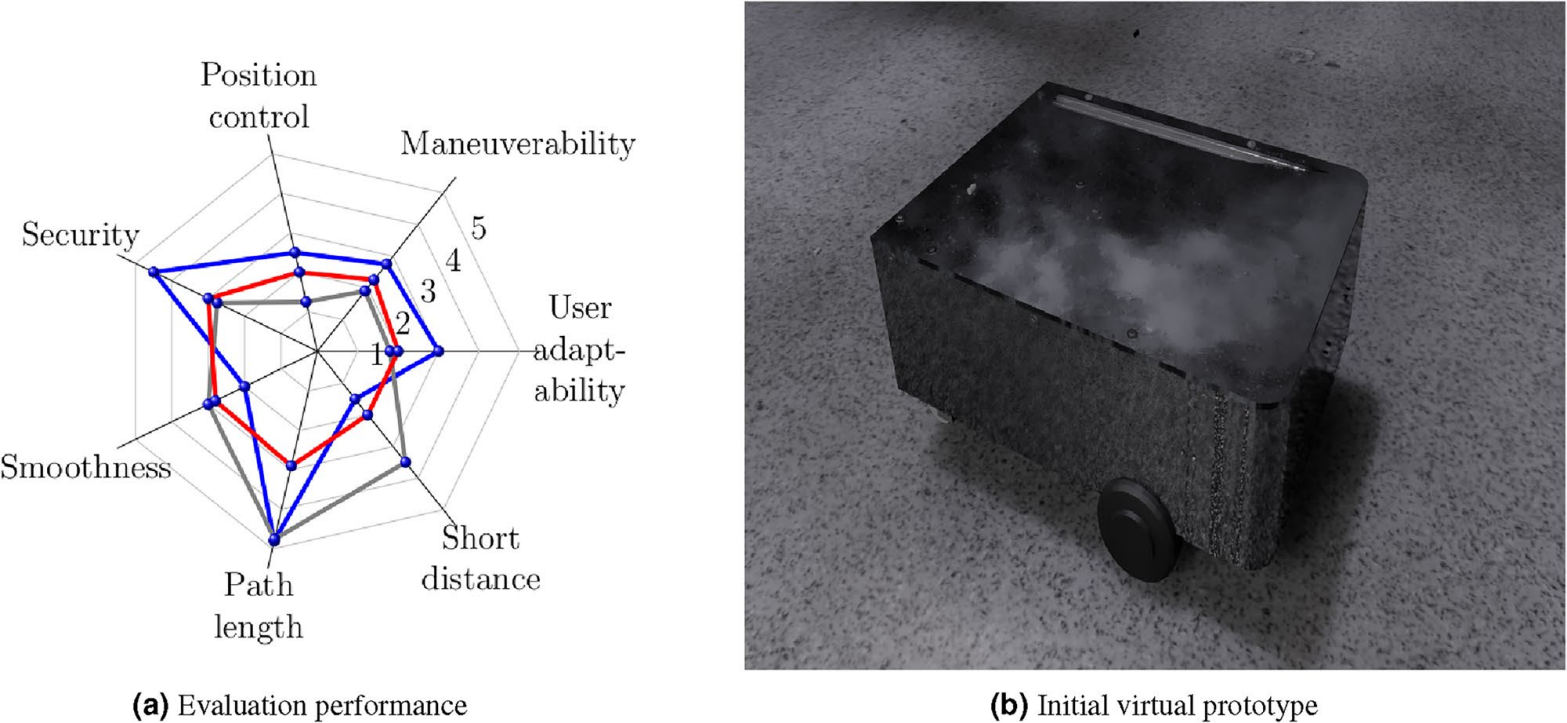
First evaluation.

In the first evaluation 16, the performance described in Fig. 16a shows that all the fields to be evaluated need to be more consistent because the initial prototype has been designed with essential elements provided by the knowledge base, as is shown in Fig. 16b. Also, the rear tires have an attraction system, and the front tires only have a guide, causing the instability of the vehicle dynamics. In this first version, the infrared sensors are only through the user’s perception. Finally, the materials were considered randomly without considering their weight or thickness dimensions.

**Figure 17:**
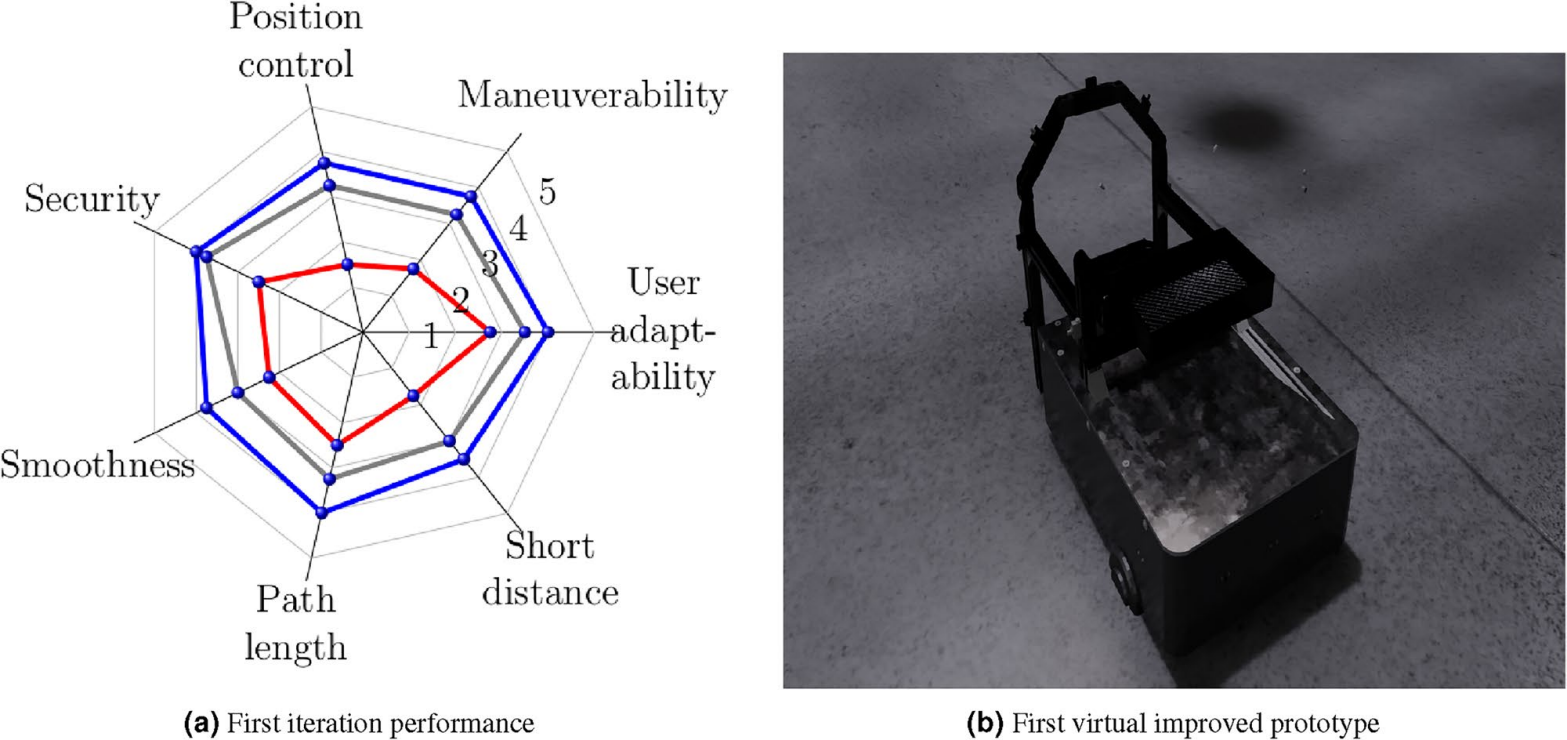
First iteration.

In the first iteration 17, Fig. 17a describes that user response improves consistently in the different items but lacks effectiveness, mainly in control and collision distance. At this stage, ultrasonic sensors were considered to support the user to avoid obstacles. A structure is added to offer stability in interacting with the agricultural environment, whose updated design is shown in Fig. 17b.

### Step 5: Implementation and evaluation

**Figure 18:**
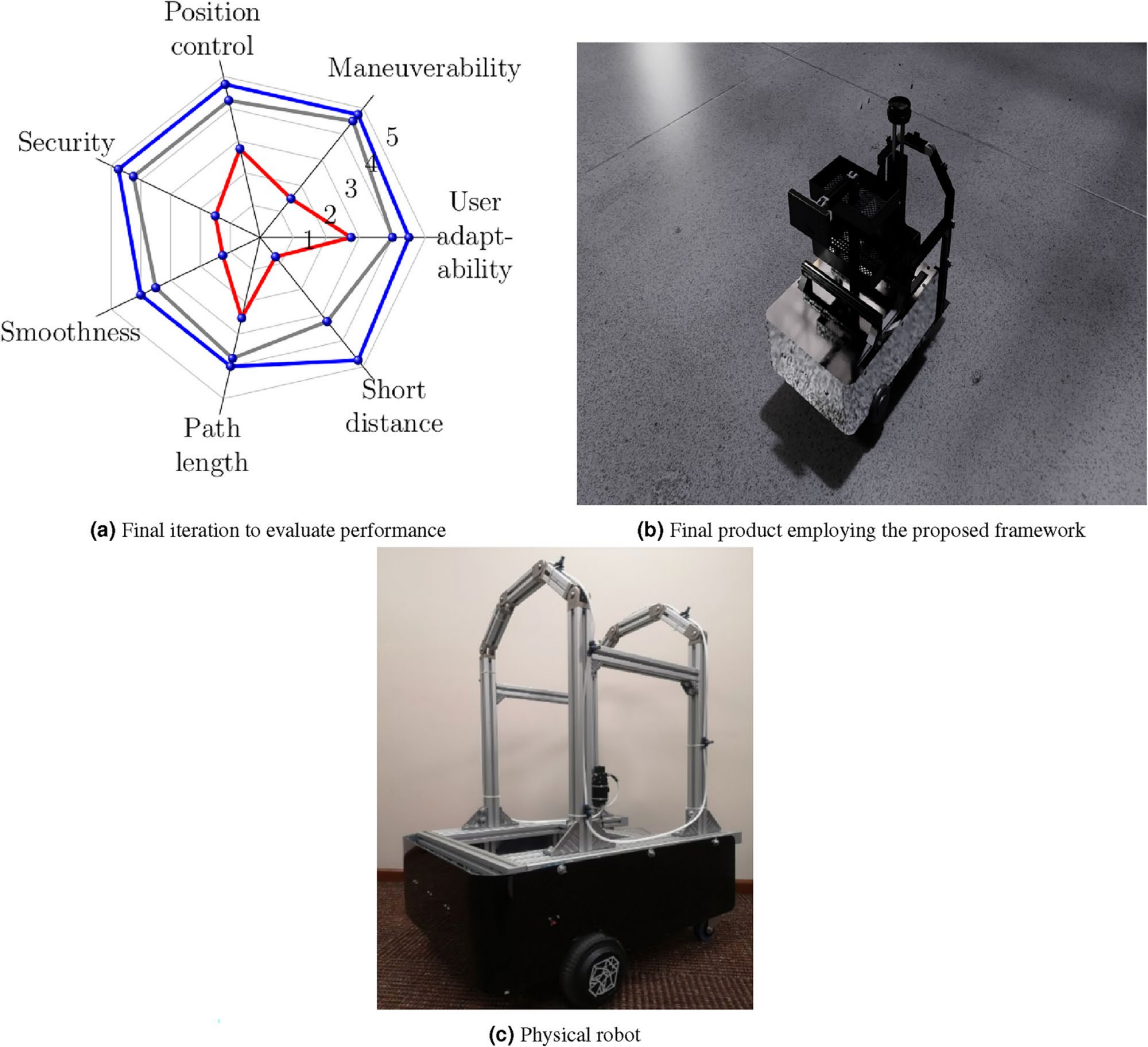
Last iteration.

In the last iteration 18, it has been considered to complement a double attraction for the rear and front. Figure 18a describes that the different items to be evaluated increased in terms of performance, offering the user a better response and control. However, at this stage, control is maintained through remote control. In addition, materials that considered real weights and dimensions, such as aluminum and fiberglass, offered better resistance and control. Motion sensors, such as the inertial measurement unit (IMU) consisting of a gyroscope, accelerometer, and magnetometer, are proposed. In addition, a camera has been installed to merge the sensors and have a better response because it reduces the error, complementing the response of the ultrasonic sensors, whose final render is shown in Fig. 18b. The navigation system is improved since it considers the states of the robot in an instant of time to describe its behavior, allowing real-time monitoring to improve the physical prototype as is shown in Fig. 18c.

## Discussion

This paper introduces a case study on developing and applying a robotic platform, specifically focusing on its potential role in controlled environments like agriculture. The contribution aims to address gaps identified in the state-of-the-art, as discussed in Table 3, which outlines the developments of various approaches reviewed in the literature confined to specific issues. In contrast, this proposal enlarges the scope of digital technologies like VR and AI to establish a framework for simulating product design, processes, and training. The comprehensive approach considers maintenance, personalized environments, user preferences, collaboration, and sustainable and circular approaches.

The research highlights the adaptability of the robot platform, demonstrating its capability to be customized for various tasks and scenarios. The platform’s integration of numerous sensors, including water, proximity, infrared, and cameras, showcases its versatility in performing a wide range of functions. However, the absence of more advanced sensors, such as thermographic cameras, is noted as a limitation that could enhance the robot’s sensing capabilities, particularly in challenging environments. The iterative design process applied throughout the study effectively enhances the robot’s features and performance, as evidenced by the three iterative phases of user adaptability, maneuverability, control, and security. While the final iteration incorporates an inertial measurement unit (IMU) and a camera, relying on remote control for navigation indicates the need for further research to evolve navigation from remote control to autonomy. The social aspect of the robot is emphasized through displayed eyes and efforts to foster operator engagement, although creating meaningful social interactions between robots and humans remains a complex challenge. This research lays a strong foundation for developing adaptable robotic systems, recognizing the importance of continuous improvement and user feedback. Future research should address identified limitations, including enhancing sensor sophistication, conducting comprehensive sustainability assessments, and refining teleoperation methods for broader real-world usability, thereby better-equipping robot platforms for diverse applications in various environments.

The virtual product design and modeling approach also streamlines the transition from design to production. Virtual simulation and optimization of workstations and manufacturing processes reduce development time and cost and enhance worker safety. This ensures that the robot’s design is innovative and practical in real-world manufacturing settings, particularly valuable in agile and responsive production scenarios like the pandemic.

In the virtual manufacturing company development stage, the framework incorporates probabilistic modeling and discrete event simulations for production line efficiency. The emphasis on a circular economy approach aligns with sustainable practices. However, it is advised to redesign the production line only when the product undergoes significant changes. In this case, two production lines have been designed.

The maintenance model discussed herein accounts for received values, offering customization options aligned with industry requirements and equipment standards. By multiplying the critical value of equipment by its associated cost, a more refined characterization of equipment is achieved, ensuring heightened quality and responsiveness within the production process. This model facilitates accurate predictions for sustaining optimal operation, employing dual evaluations to ascertain the most suitable maintenance timing.

Based on usability heuristics and deep learning, the product evaluation phase reflects a commitment to continuously improving the robot’s user experience. This iterative approach incorporates user feedback and real-world insights, making the robot more user-friendly and effective with each iteration.

**Table 3:** Identified impacts with the state-of-the-art.

Impact categories	Specification	Authors
VR and AI in the design, production, and evaluation	Use of mixed reality tools for interactive virtual prototyping to enable user participation. High-fidelity simulation environments in evaluating industrial automation. It highlights the potential of VR and AI in addressing communication in manufacturing processes. Advanced simulation and visualization techniques to optimize processes Innovation through the integration like AI, VR, and IoT	Arrighi and Mougenot (2016)^12^ Zhang et al. (2022)^25^ Lnu and Talwar (2023)^7^ Mourtzis (2023)^37^ Chu et al. (2023)^38^
Machinery maintenance, and inventory management	This paper presents a genetic algorithm-based approach to optimize preventive maintenance scheduling. Methodology for designing and implementing data-driven predictive maintenance models in manufacturing, guided by domain knowledge. Skills related to maintenance in advanced manufacturing processes.	Javanmard and Koraeizadeh (2016)^59^ Serradilla et al. (2022)^32^ Rodzalan et al. (2022)^3^
Personalized training	It emphasizes the use of VR and AI in enhancing skills and providing innovative solutions in the manufacturing sector. AI in enhancing training and skill development through interactive scenarios. Use of VR and NLP for training through interactive, conversationa scenarios	Jurczuk and Florea (2022)^4^ Joypriyanka and Surendran (2023)^8^ Lnu and Talwar (2023)^7^
Incorporation of user preferences	It emphasizes the use of VR and AI in enhancing skills and providing innovative solutions in the manufacturing sector. User experience in VR evaluations, indicating the significant influence of VR on user perception and workload.	Arrighi and Mougenot (2016)^12^ Hinricher et al. (2023)^52^
Global collaboration	Enhances productivity through the digital technologies	Chu et al. (2023)^38^
Sustainability and circular approach	It highlight the benefits of implementing reconfigurable and intelligent systems in production processes for sustainable manufacturing, using digital technologies.	Todescato et al. (2023)^15^

The proposed approaches offer many advantages within advanced manufacturing and nearshoring opportunities. They include immersive training experiences facilitated by VR, providing secure environments for workers to learn and practice skills. AI contributes by tailoring training programs to individual learning patterns, fostering personalized development. Regarding operational efficiency, AI is crucial in optimizing manufacturing processes, minimizing waste, and enhancing overall productivity. Additionally, VR expedites product development through virtual prototyping, ensuring rapid iterations.

In the development of integrating virtual activities with AI, the challenges inherent in integrating VR and AI within digital twin technology are essential for grasping the intricacies of implementing immersive and intelligent systems. These challenges encompass interoperability, equipment, data privacy and security, user interface, and ethical considerations^61,62^.

Ensuring interoperability facilitates smooth integration and communication among VR hardware and software components and AI algorithms and systems. Real-time processing presents a technical challenge, requiring a careful equilibrium between the computational needs of VR applications for real-time rendering and interaction and the demands of AI algorithms for data analysis and decision-making. This proposal advocates for designing communication between these technologies through standards tailored for handling complex rendering and data analysis tasks^63^. Another challenge of VR technology in factory environments is the potential for sensory overload and user fatigue. Prolonged use of VR headsets can lead to physical discomfort and cognitive strain, which may affect the productivity and well-being of users. Therefore, it is important to design VR experiences that are effective and ergonomically suitable for extended use in industrial applications. Additionally, the interoperability issue extends to the compatibility between different VR systems and the broader technological ecosystem within a factory. Ensuring that VR hardware and software can seamlessly connect and communicate with existing industrial systems, such as IoT devices and enterprise resource planning (ERP) software, is crucial for creating a cohesive and functional digital infrastructure. However, a sufficiently powerful computer can execute these tasks in real-time. Data privacy and security is paramount, given that VR systems collect extensive user data for immersive experiences, while AI algorithms handle sensitive information for decision-making. The proposed solution incorporates authentication mechanisms and an architecture based on Docker and containers to safeguard this sensitive information^64^. This feature guarantees that data transmitted between VR systems and AI algorithms remains confidential and shielded from unauthorized access or manipulation. User experience design is fundamental in creating intuitive interfaces for VR environments and AI-driven systems. It ensures that users navigate virtual spaces and comprehend AI-generated insights effectively. In this proposal, the interfaces in an immersive interface into the virtual environment describe the dashboard in the line of production or everyday activities. Ethical considerations arise because AI algorithms make autonomous decisions based on incomplete information, prompting concerns about accountability and transparency. Although AI is fundamental in this proposal, human experts are also involved in feedback systems to adjust the heuristics that determine decision-making^65^.

The framework’s scalability is noteworthy, accommodating robots of varying sizes, capabilities, and complexities. Lastly, VR facilitates global collaboration by enabling virtual interactions and simulations, fostering cooperation across geographical locations. Latin American countries such as Costa Rica, Colombia, Brazil, Argentina, and Chile stand to benefit significantly from nearshoring. These countries provide a range of advantages, including a highly skilled workforce, cost savings, similar time zones, cultural similarities, favorable business environments, and access to new markets, rendering them appealing destinations for nearshoring initiatives^66^. Moreover, Europe, particularly Eastern European nations like Poland, Ukraine, Romania, the Czech Republic, and Hungary, is emerging as a popular choice for nearshoring IT services. This trend is fueled by factors such as a skilled talent pool, cost-effectiveness, cultural affinity, and favorable business environments^67^. Nearshoring to Europe offers benefits such as access to a diverse talent pool, competitive pricing, ease of communication, proximity in time zones, and adherence to strict data protection laws, making it an enticing option for companies seeking to outsource software development projects.

The framework aids companies in utilizing the skilled labor available in nearshoring regions, which is crucial for optimizing production processes and fostering innovation. Cost reduction, a key driver of nearshoring, is another area where the framework proves invaluable. It offers a structured methodology for companies to realize financial benefits while upholding quality standards and effectively navigating the economic landscapes of nearshoring destinations. Moreover, the framework emphasizes the importance of cultural affinity and understanding the business environment of the host country. This understanding is vital for building strong local relationships, adapting to business practices, complying with regulatory requirements, ensuring smoother operations, and minimizing risks. The adaptability of the framework to different regions and market conditions underscores its utility, providing companies with a tool to expand or adjust their nearshoring strategies in response to changing business dynamics. Additionally, it supports enhancing supply chain management by promoting closer geographic proximity and better alignment with local suppliers and partners.

This proposed framework is a practical guide for manufacturing companies to exploit nearshoring opportunities fully. Its broad applicability across different geographical and economic contexts makes it a strategic asset for companies seeking to improve performance and secure sustainable growth in the global manufacturing landscape.

### Limitations

The approach presented by the authors focuses on developing virtual simulations and models that generate ideal solutions. However, a significant challenge arises when attempting to implement these developments in the real world. The primary advantage of creating a virtual representation is the ability to identify necessary variables and characteristics to define elements that describe products and are essential for the production line. Furthermore, simulation allows for obtaining data to develop stochastic representations that realistically describe behavior in the real world, thereby contributing to reducing costs and design times, as concluded in this work. The model for determining maintenance is divided into two periods: short-term (less than a year) and long-term (more than a year). However, by employing virtual models, it is possible to consider more variables to determine more precise maintenance and make decisions since there are processes in the production chain that require less maintenance in terms of period. The current proposal is contingent on the level of automation in manufacturing. Criteria such as factory capacity, synchronization, optimization, and reliability are crucial. Factory features, such as material and logistical synchronization, physical synchronization to maintain adequate communication between different sectors, optimization describing productive and non-productive aspects of a system, and the ability to overcome failures using sensors and cameras for enhanced control and adaptability, impact the proposal’s performance. The iterative process demonstrates a commitment to continuous improvement, ensuring the robot remains relevant and effective in dynamic environments and determining the necessary maintenance to keep good performance in the production line based on Genetic Algorithms.

The integration of VR and AI technologies presents several challenges. Substantial financial resources are required to acquire hardware, software, and training, contributing to the overall cost. Furthermore, developing, implementing, and maintaining these technologies demand specific technical skills, adding a layer of technical complexity. While VR simulations offer a secure training environment, their limitations in replicating real-world scenarios restrict the practical experience gained by workers.

## Conclusion

This paper has delved into developing and applying a comprehensive framework for virtual commissioning in robotics, specifically focusing on addressing challenges posed by the COVID-19 pandemic. The proposed framework has been demonstrated through an in-depth case study, highlighting its efficacy in creating agile and adaptable robotic solutions. The framework’s multi-dimensional approach, encompassing Sensing, Smart capabilities, Sustainability, and Social interaction (S4), forms a crucial foundation for designing robots capable of versatile tasks across diverse environments. This adaptability is vital during crises like the pandemic, where robotics is crucial in maintaining public safety and ensuring continuity across various sectors. The detailed analysis of each step in the case study provides valuable insights into applying the framework to develop innovative products, like low-cost robots, and adapting them to address specific challenges, such as those posed by the COVID-19 pandemic. Integrating virtual reality and simulations into manufacturing illustrates how technology can streamline development, improve efficiency, and enhance the end-user experience. This case study serves as a valuable reference for future projects to develop and commission advanced robotic systems.

The phases of virtual product design and modeling, virtual manufacturing company development, and product evaluation underscore the practicality and efficiency of the framework. The ability to virtually simulate workstations and production processes expedites development and enhances worker safety and resource efficiency. Furthermore, the inclusion of probabilistic modeling, discrete event simulations, and a circular economy approach emphasizes a commitment to sustainability in robotic production.

The iterative approach to product evaluation, guided by usability heuristics and deep learning, ensures continuous improvement in the user experience, which is crucial in human–robot interaction. The framework’s user adaptability, maneuverability, and user-friendly interface provide a solid foundation for effective communication and interaction in real-world applications.

The implementation and evaluation phase highlights the forward-thinking nature of the framework, introducing advanced motion sensors and cameras to enhance control and adaptability. The iterative process demonstrates a dedication to ongoing improvement, ensuring the robot remains relevant and effective in dynamic environments.

Hence, future efforts should be directed toward transitioning from virtual commissioning to real-world implementation. Field trials and deployment of robots, such as the ROBOCOV, equipped with features developed through this framework, would yield invaluable insights into the practical challenges and opportunities arising in real-world scenarios. Observing how these robots operate in healthcare settings, manufacturing facilities, and agricultural contexts can lead to significant refinements and optimizations. Further research into advanced machine learning and artificial intelligence algorithms tailored to robotic systems can enable them to learn from their experiences and surroundings, resulting in more intuitive, context-aware behavior encompassing adaptive task planning, decision-making, and enhanced environmental understanding. Scalability is another critical aspect that warrants further exploration, with research focusing on methods to adapt the framework to accommodate robots of varying sizes, capabilities, and complexities, making it applicable to a broader range of robotic systems.

## Data Availability

The datasets used and/or analysed during the current study available from the corresponding author on reasonable request.

## References

[1] IADB. Iadb | nearshoring can add annual \$78 bln in exports from latin america and caribbean, (accessed 25 October 2023). https://www.iadb.org/en/news/nearshoring-can-add-annual-78-bln-exports-latin-america-and-caribbean (2022).

[2] FitchRatings. Nearshoring is a multi-year tailwind for mexican corporate credits, (accessed 25 October 2023). https://www.fitchratings.com/research/corporate-finance/nearshoring-is-multi-year-tailwind-for-mexican-corporate-credits-07-08-2023 (2023).

[3] Rodzalan SA (2022). An investigation of present and future work skills in industry 4.0: Systematic literature review. J. Adv. Res. Appl. Sci. Eng. Technol..

[4] Jurczuk A, Florea A (2022). Future-oriented digital skills for process design and automation. Hum. Technol..

[5] Li L (2022). Reskilling and upskilling the future-ready workforce for industry 4.0 and beyond. Inf. Syst. Front..

[6] Akyazi T, del Val P, Goti A, Oyarbide A (2022). Identifying future skill requirements of the job profiles for a sustainable European manufacturing industry 4.0. Recycling.

[7] Lnu, H. & Talwar, R. Umeed: VR game using NLP models and latent semantic analysis for conversation therapy for people with speech disorders. In *Artificial Intelligence, NLP , Data Science and Cloud Computing Technology*, (Academy & Industry Research Collaboration, 2023) 10.5121/csit.2023.131408

[8] Joypriyanka, M. & Surendran, R. Priority experience replay DQN for training an agent in virtual reality game for kids with paraplegia. In *2023 2nd International Conference on Applied Artificial Intelligence and Computing (ICAAIC)* (IEEE, 2023). 10.1109/icaaic56838.2023.10141031.

[9] Zhang J, Wang P, Zuo M, Li Y, Xu Z (2015). Automatic assembly simulation of product in virtual environment based on interaction feature pair. J. Intell. Manuf..

[10] Wenning M, Backhaus AA, Adlon T, Burggräf P (2022). Testing the reliability of monocular obstacle detection methods in a simulated 3d factory environment. J. Intell. Manuf..

[11] Fang W, Zhang T, Chen L, Hu H (2023). A survey on HoloLens AR in support of human-centric intelligent manufacturing. J. Intell. Manuf..

[12] Arrighi P-A, Mougenot C (2016). Towards user empowerment in product design: A mixed reality tool for interactive virtual prototyping. J. Intell. Manuf..

[13] Wei S, Nourelfath M, Nahas N (2023). Analysis of a production line subject to degradation and preventive maintenance. Reliab. Eng. Syst. Saf..

[14] Geurtsen M, Didden JB, Adan J, Atan Z, Adan I (2023). Production, maintenance and resource scheduling: A review. Eur. J. Oper. Res..

[15] Todescato M (2023). Sustainable manufacturing through application of reconfigurable and intelligent systems in production processes: A system perspective. Sci. Rep..

[16] van Hassel E (2022). Reconsidering nearshoring to avoid global crisis impacts: Application and calculation of the total cost of ownership for specific scenarios. Res. Transp. Econ..

[17] Guedes JF, Pereira L (2016). Proximity Offshoring Generating Considerable Savings with No Significant Increase of Risks or Losses in Quality-Nearshoring Playing a Key Role on a Business Transformation Program.

[18] Kanesvaran, S. The industrial metaverse: What it is and what it means for you, (accessed 30 December 2023). https://blog.se.com/sustainability/2023/03/07/the-industrial-metaverse-what-it-is-and-what-it-means-for-you/ (2023).

[19] SIEMENS. Go vr for fully immersive design experience, (accessed 30 December 2023). https://www.plm.automation.siemens.com/global/pt/products/collaboration/virtual-reality.html (2022).

[20] NVIDIA. A new era of immersive digital twin technology, (accessed 30 December 2023). https://www.nvidia.com/en-us/omniverse/digital-twins/siemens/ (2023).

[21] Méndez, J. I. *et al.* S4 product design framework: A gamification strategy based on type 1 and 2 fuzzy logic. In *International Conference on Smart Multimedia*, 509–524 (Springer, 2019).

[22] Molina A, Ponce P, Miranda J, Cortés D (2021). Enabling Systems for Intelligent Manufacturing in Industry 4.0.

[23] Kumar S, Mohan S, Skitova V (2023). Designing and implementing a versatile agricultural robot: A vehicle manipulator system for efficient multitasking in farming operations. Machines.

[24] Kharzhevskyi A, Horiashchenko S, Kharzhevskyi V (2022). Justification of the design and parameters of the wheels during the design process of the chasis of a robotic platform using solidworks simulation. Herald Khmelnytskyi Natl. Univ. Tech. Sci..

[25] Zhang Z (2022). A high-fidelity simulation platform for industrial manufacturing by incorporating robotic dynamics into an industrial simulation tool. IEEE Robot. Autom. Lett..

[26] Stan L, Nicolescu AF, Pupăză C, Jiga G (2022). Digital twin and web services for robotic deburring in intelligent manufacturing. J. Intell. Manuf..

[27] Molotla O, Peña-Cabrera JM, Lomas-Barrie V (2023). Configurable hybrid integral manufacturing platform: Subtractive-additive process with industrial robot arm, proof of concept results. IEEE Lat. Am. Trans..

[28] Kim T, Park Y-L (2023). Robotic platform for automatic alignment and placement of fabric patterns for smart manufacturing in garment industry. Int. J. Precis. Eng. Manuf..

[29] Park J, Han C, Jun MBG, Yun H (2023). Autonomous robotic bin picking platform generated from human demonstration and YOLOv5. J. Manuf. Sci. Eng..

[30] Formoso, O. *et al.* MMIC-i: A robotic platform for assembly integration and internal locomotion through mechanical meta-material structures. In *2023 IEEE International Conference on Robotics and Automation (ICRA)* (IEEE, 2023). 10.1109/icra48891.2023.10161263.

[31] Vijayakumar K (2022). On blockchain technology and machine learning algorithms in concurrent engineering. Concurr. Eng..

[32] Serradilla O, Zugasti E, de Okariz JR, Rodriguez J, Zurutuza U (2022). Methodology for data-driven predictive maintenance models design, development and implementation on manufacturing guided by domain knowledge. Int. J. Comput. Integr. Manuf..

[33] Starikov, A. *et al.* Virtual furniture design bureau: Distributed design in multi-agent environment using cloud technologies. In *Proceedings of the Russian Conference on Digital Economy and Knowledge Management (RuDEcK 2020)*(Atlantis Press, 2020). 10.2991/aebmr.k.200730.118.

[34] Effendi R, Efendi Y (2020). Design and implementation of virtual local area network network at pt. eds manufacturing Indonesia. J. Innov. Future Technol. (IFTECH).

[35] Marconi M, Manieri S, Germani M, Raffaeli R (2018). A digitally-enabled integrated approach to design and manufacture shoe lasts. Comput. Aided Des. Appl..

[36] Zhou C (2016). Integration of advanced simulation and visualization for manufacturing process optimization. JOM.

[37] Mourtzis D (2023). The metaverse in industry 5.0: A human-centric approach towards personalized value creation. Encyclopedia.

[38] Chu, C.-H., Baroroh, D. K., Pan, J.-K. & Chen, S.-M. An exemplary case of industrial metaverse: Engineering product demonstration using extended reality technologies. *Int. J. Precis. Eng. Manuf. Smart Technol.* **1**, 243–250. 10.57062/ijpem-st.2023.0038 (2023).

[39] Muthmainnah, Yakin, A. A. & Seraj, P. M. I. Impact of metaverse technology on student engagement and academic performance: The mediating role of learning motivation. *Int. J. Comput. Inf. Manuf. (IJCIM)* **3**, 10–18. 10.54489/ijcim.v3i1.234 (2023).

[40] Wang J, Suo J, Song Z, Li WJ, Wang Z (2023). Nanomaterial-based flexible sensors for metaverse and virtual reality applications. Int. J. Extreme Manuf..

[41] Li K (2023). Toward ubiquitous semantic metaverse: Challenges, approaches, and opportunities. IEEE Internet Things J..

[42] Deris S, Omatu S, Ohta H, Shaharudin Kutar L, Abd Samat P (1999). Ship maintenance scheduling by genetic algorithm and constraint-based reasoning. Eur. J. Oper. Res..

[43] Sortrakul N, Nachtmann H, Cassady C (2005). Genetic algorithms for integrated preventive maintenance planning and production scheduling for a single machine. Comput. Ind..

[44] Einabadi B, Mahmoodjanloo M, Baboli A, Rother E (2023). Dynamic predictive and preventive maintenance planning with failure risk and opportunistic grouping considerations: A case study in the automotive industry. J. Manuf. Syst..

[45] Hivarekar, N., Jadav, S., Kuppusamy, V., Singh, P. & Gupta, C. Preventive and predictive maintenance modeling. In *2020 Annual Reliability and Maintainability Symposium (RAMS)* 1–6. 10.1109/RAMS48030.2020.9153636 (2020).

[46] Geissdoerfer M, Vladimirova D, Evans S (2018). Sustainable business model innovation: A review. J. Clean. Prod..

[47] Deng, Q., Franke, M., Lejardi, E. S., Rial, R. M. & Thoben, K.-D. Development of a digital thread tool for extending the useful life of capital items in manufacturing companies—An example applied for the refurbishment protocol. In *2021 26th IEEE International Conference on Emerging Technologies and Factory Automation (ETFA )* 1–8. 10.1109/ETFA45728.2021.9613143 (2021).

[48] Meijdam, H. *Circular Economy. From Wish to Practice*, vol. 1 (Council for the Environment and Infrastructure, 2015).

[49] Bortolini M, Calabrese F, Galizia FG, Regattieri A (2023). A two-step methodology for product platform design and assessment in high-variety manufacturing. Int. J. Adv. Manuf. Technol..

[50] Wang Y, Liu Q (2023). A virtual evaluation system for product designing using virtual reality. Soft. Comput..

[51] Palacios-Ibáñez A, Felip-Miralles F, Galán J, García-García C, Contero M (2023). Consumer subjective impressions in virtual reality environments: The role of the visualization technique in product evaluation. Electronics.

[52] Hinricher N, König S, Schröer C, Backhaus C (2023). Effects of virtual reality and test environment on user experience, usability, and mental workload in the evaluation of a blood pressure monitor. Front. Virtual Real..

[53] Felip F, Galán J, Contero M, García-García C (2023). Touch matters: The impact of physical contact on haptic product perception in virtual reality. Appl. Sci..

[54] Tomashenko, N. *et al.* The voiceprivacy 2020 challenge evaluation plan, 10.48550/ARXIV.2205.07123 (2022).

[55] Ponce P (2022). S4 features and artificial intelligence for designing a robot against COVID-19—Robocov. Future Internet.

[56] Ponce P, Balderas D, Peffer T, Molina A (2018). Deep learning for automatic usability evaluations based on images: A case study of the usability heuristics of thermostats. Energy Build..

[57] Gravina, D., Liapis, A. & Yannakakis, G. N. Constrained surprise search for content generation. In *2016 IEEE Conference on Computational Intelligence and Games (CIG)* 1–8. 10.1109/CIG.2016.7860408 (2016).

[58] Ceron-Lopez, A. E., Ranjan, R. & Koganti, N. Realism assessment for synthetic images in robot vision through performance characterization. In *2022 IEEE/RSJ International Conference on Intelligent Robots and Systems (IROS)* 13089–13096. 10.1109/IROS47612.2022.9982192 (2022).

[59] Javanmard H, Koraeizadeh AA-W (2016). Optimizing the preventive maintenance scheduling by genetic algorithm based on cost and reliability in national Iranian drilling company. J. Ind. Eng. Int..

[60] Morseletto P (2020). Targets for a circular economy. Resour. Conserv. Recycl..

[61] He Q (2024). From digital human modeling to human digital twin: Framework and perspectives in human factors. Chin. J. Mech. Eng..

[62] Lee S, Kim S, Roh B (2024). Mixed reality virtual device (MRVD) for seamless MR-IoT-digital twin convergence. Internet Things.

[63] Elkoura, G. *et al.* A deep dive into universal scene description and hydra. In *ACM SIGGRAPH 2019 Courses*, SIGGRAPH ’19, (ACM, 2019). 10.1145/3305366.3328033.

[64] Lima GLD, Aguiar MSD (2024). Towards a docker-based architecture for open multi-agent systems. IAES Int. J. Artif. Intell. (IJ-AI).

[65] Adam M, Wessel M, Benlian A (2020). Ai-based chatbots in customer service and their effects on user compliance. Electron. Mark..

[66] Yakovlev P (2023). Model nearshoring—The concept of the new economy of the Latin America. World Econ. Int. Relat..

[67] Keller F, Zoller-Rydzek B (2019). European nearshoring index–is eastern Europe attractive for swiss it firms?. Cent. Eur. Bus. Rev..

